# Ruthenium Polypyridine Complexes Combined with Oligonucleotides for Bioanalysis: A Review

**DOI:** 10.3390/molecules190811933

**Published:** 2014-08-11

**Authors:** Shuyu Zhang, Yubin Ding, Hui Wei

**Affiliations:** Department of Biomedical Engineering, College of Engineering and Applied Sciences, Nanjing University, Nanjing 210093, China; E-Mails: zhangshuyu1990_cn@163.com (S.Z.); ybding@nju.edu.cn (Y.D.)

**Keywords:** aptamer, bioanalysis, DNA detection, DNAzyme, electrochemiluminescence, electrochemistry, functional nanomaterials, ruthenium complexes

## Abstract

Ruthenium complexes are among the most interesting coordination complexes and they have attracted great attention over the past decades due to their appealing biological, catalytic, electronic and optical properties. Ruthenium complexes have found a unique niche in bioanalysis, as demonstrated by the substantial progress made in the field. In this review, the applications of ruthenium complexes coordinated with polypyridine ligands (and analogues) in bioanalysis are discussed. Three main detection methods based on electrochemistry, electrochemiluminescence, and photoluminscence are covered. The important targets, including DNA and other biologically important targets, are detected by specific biorecognition with the corresponding oligonucleotides as the biorecognition elements (*i.e.*, DNA is probed by its complementary strand and other targets are detected by functional nucleic acids, respectively). Selected examples are provided and thoroughly discussed to highlight the substantial progress made so far. Finally, a brief summary with perspectives is included.

## 1. Introduction

**Figure 1:**
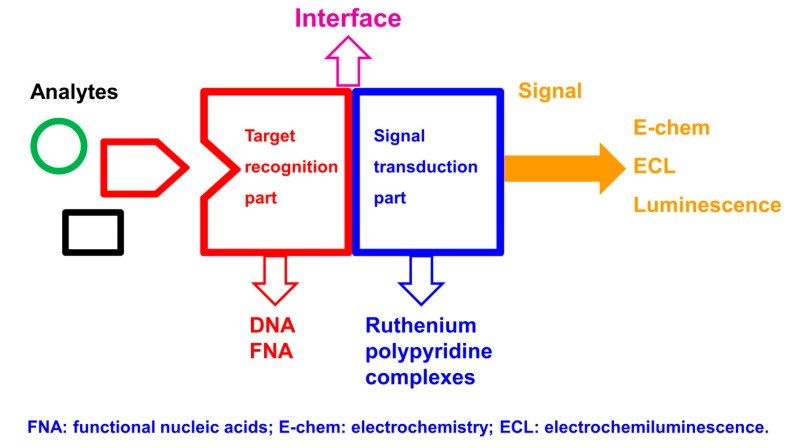
Bioanalytical platform using ruthenium polypyridine complexes (and analogues) as signal transduction elements and oligonucleotides as target recognition elements.

Since Alfred Werner’s pioneering work more than one hundred years ago, substantial progress has been made in the field of coordination chemistry. In the last few decades, the research of coordination chemistry has been extended to numerous interesting and emerging fields, such as analytical chemistry [1,2], biomimetic chemistry [3], catalysis [4,5], cell biology [6], functional nanomaterials [7,8,9,10,11,12,13,14,15,16,17,18], medical and clinical chemistry [19,20,21], and supramolecular chemistry [22,23,24,25]. A huge number of coordination complexes have been synthesized using different metal ions and ligands. Among them, ruthenium complexes coordinated with polypyridine ligands (and analogues) have received considerable attention due to their unique biological, catalytic, electronic and optical properties, and their emerging applications in bioanalysis, bioimaging, solar cells, and organic light-emitting diodes [26,27,28,29,30,31,32]. Since many comprehensive reviews have been devoted to ruthenium polypyridine complexes and their various applications [27,28,30,31,33], this review will specifically focus on the recent progress in the field of bioanalysis, using ruthenium polypyridine complexes (and analogues) as signal transduction elements and oligonucleotides as target recognition elements (Figure 1). DNA detection based on Watson-Crick base-pairing recognition and the detection of other important targets with functional nucleic acids (such as aptamer and DNAzymes) as biorecognition elements will be highlighted. Three main detection methods,* i.e.*, electrochemistry, electrochemiluminescence (ECL), and photoluminescence, will be extensively discussed.

We begin by introducing electrochemical DNA detection based on ruthenium polypyridine complexes-mediated catalytic oxidation of DNA bases (mainly guanine) in Section 2. The use of ruthenium complexes as redox active tags is also covered in Section 2. The use of ruthenium polypyridine complexes as luminophores, ECL detection of DNA and other important analytes with functional nucleic acids is covered in Section 3. In Section 4, the photoluminscence detection method is discussed, highlighting the “light-switch” effect of ruthenium complexes and their application in DNA analysis. Finally, a brief summary with perspectives is included. 

## 2. Electrochemical Methods

Highly sensitive and selective DNA detection is of great importance in numerous areas, including clinical diagnostics, environmental monitoring, forensic chemistry, gene analysis, pharmaceutical industry, and homeland security [34,35,36]. Thus, great efforts have been made for DNA detection. Among many methods developed, electrochemical methods have shown the advantages of high sensitivity, excellent selectivity, low-cost, portability, ready for integration and miniaturization, and easy-to-operate properties [34,37,38,39,40].

In the early days, researchers explored the direct reduction or oxidation of DNA for DNA sensing [41,42]. Direct electrochemical DNA sensing approaches, however, were inherently not highly sensitive. This was because that the signal to noise ratio obtained this way was often low. Additionally, significant background current was often obtained due to the high potentials required for direct reduction or oxidation of DNA.

**Figure 2:**
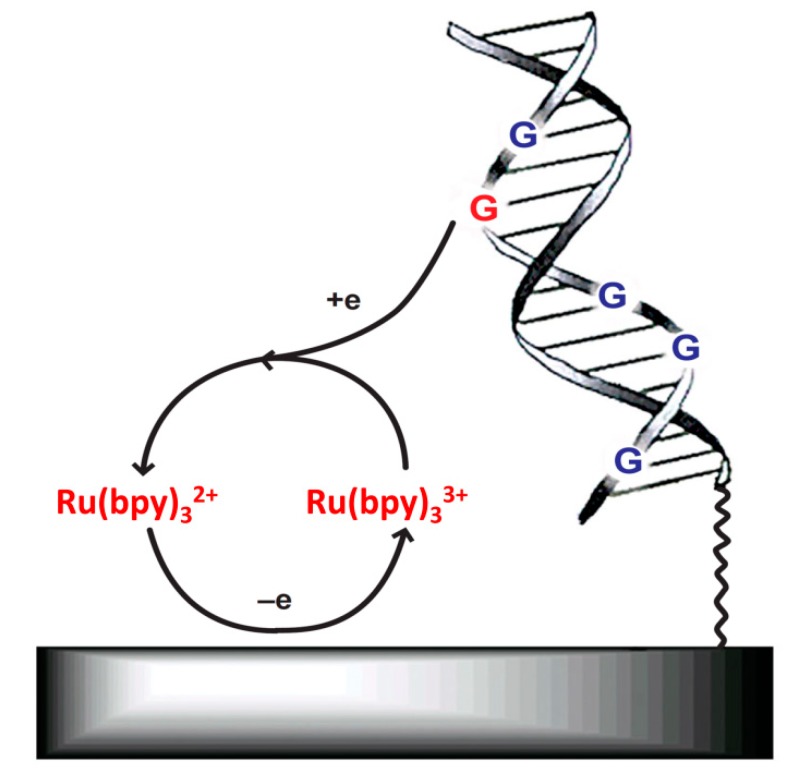
Using Ru(bpy)_3_^2+^ as a redox-active mediator to electrochemically detect DNA. Adapted with permission from ref. [34], copyright (2003) Nature Publishing Group.

**Figure 3:**
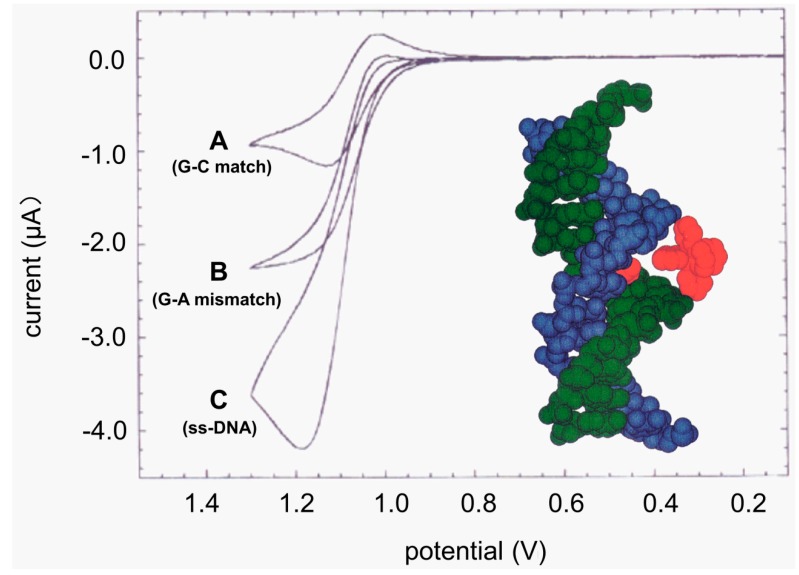
Using Ru(bpy)_3_^2+^ as a redox-active mediator to electrochemically distinguish native double helix, its single-base mismatch counterpart and the corresponding ssDNA from one another. Inset: computer model showing the interaction between Ru(bpy)_3_^2+^ and a guanine residue in a double helix (both in red). Adapted with permission from ref. [44], copyright (1995) American Chemical Society.

By using redox-active mediators, such as tris(2,2'-bipyridine)ruthenium(II) (Ru(bpy)_3_^2+^) to bring the electrons from DNA (mainly guanine residues for oxidation) to the electrode surface, the obstacles mentioned above can be overcome (Figure 2) [43]. An interesting method was developed by Thorp and coworkers [44,45,46,47,48,49,50,51,52]. As shown in Figure 2 and Equations (1) and (2), under the applied potential, Ru(bpy)_3_^2+^ is first oxidized to Ru(bpy)_3_^3+^ on an electrode. The guanine residues of the DNA then reduce Ru(bpy)_3_^3+^ to regenerate Ru(bpy)_3_^2+^, thus forming a catalytic cycle. Under a given concentration of Ru(bpy)_3_^2+^, the current is proportional to the concentration of guanine residues (and thus the concentration of target DNA). In the seminal study, it was found that the electron transfer from guanine residues to Ru(bpy)_3_^3+^ must be tuned through solvent since the native double helix structure precluded the direct interaction between them (Figure 3, inset) [44]. The mismatch (or complete denaturation) in a double helix decreased the tunneling distance and thus enhanced the electron transfer efficiency and guanine oxidation. As shown in Figure 3, using this method, they were able to distinguish native double helix, its single-base mismatch counterpart and the corresponding single-stranded DNA (ssDNA) from one another:

Ru(bpy)_3_^2+^ → Ru(bpy)_3_^3+^ + e^−^ (1)

Ru(bpy)_3_^3+^ + DNA → DNA_ox_ + Ru(bpy)_3_^2+^ (2)

In the subsequent studies, Thorp and coworkers demonstrated that the background signals from a capture DNA strand could be minimized by replacing guanine residues with inosine residues in the capture DNA strand [45]. Inosine could still recognize cytosine, but was less active than guanosine by nearly three orders of magnitude [45]. With the inosine-substituted capture DNA strands, the polymerase chain reaction (PCR) products of HIV, herpes simplex II virus (HSV) and *Clostridium perfringens* were successfully determined, respectively. Moreover, with the same method, mRNA of overexpressed Rak nuclear tyrosine kinase from breast tumor was quantitatively measured after competitive reverse transcription-PCR (RT-PCR) amplification [52].

The Ru(bpy)_3_^2+^-mediated DNA catalytic oxidation was also employed to detect chemically induced DNA damage [53]. Rusling* et al.* constructed a sensing platform by layer-by-layer assembly of poly(diallyldimethylammonium chloride) (PDDA) and double-stranded DNA (dsDNA) on a graphite electrode. Before incubating with a DNA damaging agent, such as styrene oxide, the electrochemical signal from Ru(bpy)_3_^2+^-mediated DNA catalytic oxidation was obtained. After incubating with styrene oxide, an enhanced electrochemical signal was observed. Interestingly, the electrochemical signals increased linearly with the increase of incubation time. For nonreactive toluene, only minor electrochemical signal changes were detected before and after incubation [53]. The redox active polymer [Ru(bpy)_2_(PVP)_10_](ClO_4_)_2_ (where PVP is poly(4-vinylpyridine)) has also been employed for catalytic oxidation of DNA. Recently, Rusling and coworkers reported a simple and rapid microfluidic array for reactive metabolite screening [54]. The eight-electrode array was fabricated by assembling DNA films onto screen-printed carbon electrodes. If the reactive metabolites, secreted by cytochrome P450 enzyme in rat liver microsomes, were harmful to DNA and caused its damage, enhanced catalytic electrochemical signals would be obtained. Due to the high throughput and high sensitivity, the proposed method may be used for toxicity screening in the future [54]. Compared with free Ru(bpy)_3_^2+^ in solution, the incorporation of Ru(bpy)_3_^2+^ into a polymer matrix, like [Ru(bpy)_2_(PVP)_10_](ClO_4_)_2_, reduces the reagent use and allows the fabrication of solid state biosensors [46,55].

An interesting ruthenium complex,* i.e.*, [Ru(dmbpy)_2_(PIND)_2_]^2+^ (where dmbpy is 4,4'-dimethyl-2,2'-pyridine and PIND is N,N'-bis(3-propylimidazole)-1,4,5,8-naphthalenediimide), was synthesized by Xie and coworkers [56]. [Ru(dmbpy)_2_(PIND)_2_]^2+^ exhibited ideal Nernstian behavior, demonstrating good electrochemical properties. The hypochromism and red-shift of [Ru(dmbpy)_2_(PIND)_2_]^2+^ after adding dsDNA confirmed the intercalative interaction between the ruthenium complex and dsDNA. It suggested that the two PIND ligands bound to the dsDNA in a threading intercalation mode, while the cationic parts ([Ru(dmbpy)_2_]^2+^) strengthened the intercalative interaction via electrostatic attraction. The ruthenium complex was then used as an electroactive indicator for DNA sensing. The probe ssDNA and its complementary target ssDNA formed DNA duplex, which could intercalate with [Ru(dmbpy)_2_(PIND)_2_]^2^^+^ and produce a distinct voltammetric peak. On the other hand, the presence of non-complementary ssDNA only produced negligible signals. The sensing method had a dynamic range of 1.5 to 300 nM and a detection limit of 0.80 nM [56]. No mismatch detection was performed. Since [Ru(dmbpy)_2_(PIND)_2_]^2+^ was also ECL active, it was also used for ECL detection of DNA. Compared with the electrochemical method, the ECL method showed 2,000-fold sensitivity enhancement (see Section 3 for more information) [56]. Other intercalative type ruthenium complexes were also developed for DNA assay [57].

**Figure 4:**
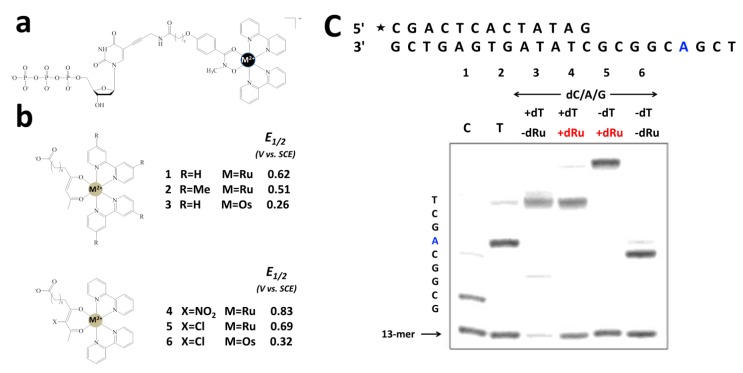
Incorporation of redox active metal complexes into nucleotides for PCR. (**a**) structure of redox-active dTTP analogue; (**b**) structures of metal complexes to be incorporated into nucleotides; (**c**) PCR reaction with electrochemical active nucleotides. Adapted with permission from ref. [58], copyright (2002) American Chemical Society.

Usually redox active probes, such as Ru(bpy)_3_^2+^, are chemically conjugated onto a probe ssDNA for biosensing. Tor and coworkers demonstrated that redox active probes could be introduced into nucleotides for the first time [58,59]. Based on thoughtful design, modified nucleotides with electrochemical activities were synthesized (Figure 4). More, they could be incorporated into target ssDNA by DNA polymerase. To be compatible with the PCR reaction, several criteria were considered to design and synthesize the modified nucleotides. First, the E_1/2_ of the inserted metal complex should be within a redox potential window, in which DNA bases may not be oxidized or reduced; second, the modified nucleotides should be stable; third, they can be electrophoretically separated. Since Ru(bpy)_3_^2+^ has an E_1/2_ of 1.25 V (vs SCE), one of the polypyridine ligand was replaced with a negatively charged ligand to lower the oxidation potential. More, by using different metal centers (such as Ru vs Os) and polypyridine derivatives, tunable E_1/2_ could be obtained (Figure 4b). Thus, the nucleotides inserted with the redox active metal complexes could be even used for multiplex sensing and diagnosis. It was demonstrated that the modified nucleotides were successfully incorporated into the target ssDNA at designed sites by PCR reaction (Figure 4c) [58]. 

**Figure 5:**
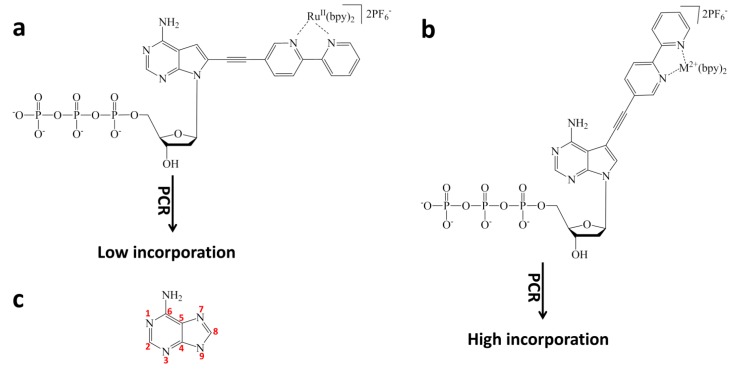
Incorporation of redox active metal complexes into purine bases for PCR. (**a**) incorporation in C-8 position; (**b**) incorporation in N-7 position; (**c**) the numbering of adenine. (**a**) adapted with permission from ref. [60], copyright (2007) John Wiley and Sons; (**b**) adapted with permission from ref. [63], copyright (2009) John Wiley and Sons.

Hocek and coworkers reported the first examples of inserting ruthenium complexes into purine bases (Figure 5) [60,61,62,63]. The linkage of the ruthenium complex to C-8 position of a purine base was achieved by aqueous crossing linking. However, these 8-substituted purine bases could not be efficiently introduced into target ssDNA by PCR reaction due to the destabilizing *syn*-conformation [61,64]. Later, they showed that the linkage of the ruthenium complex to N-7 position of a purine base could be realized by aqueous crossing linking of 7-iodo-7-deaza-2'-deoxyadenosine and boric acid (or acetylene) modified metal complexes [61,63]. More, the metal complexes containing nucleotides could be incorporated into target ssDNA by PCR at high yield (Figure 5b) [63]. The electrochemical studies showed that the signal of labeled Ru(bpy)_3_^2+^ (1.1–1.25 V* vs.* SCE) was overlapped by the signal of guanine bases (1.1 V* vs.* SCE). So it may not be suitable for electrochemical analysis with the Ru(bpy)_3_^2+^ labels. However, the electrochemical signal of labeled Os(bpy)_3_^2+^ (0.75 V* vs.* SCE) was not influenced by DNA base oxidation, making it ideal electrochemical label. When combined with other redox active labels such as ferrocene, aminophenyl, and nitrophenyl moieties, multiple “colors” of DNA by PCR and multiplex electrochemical DNA detection were successfully accomplished [63].

## 3. Electrochemiluminescent Methods

Derived from the simple and inexpensive platform offered by electrochemical DNA sensing, a more sensitive analytical strategy has been proposed, namely, the ECL-based DNA detection. ECL based on Ru(bpy)_3_^2+^ and its analogues has been widely used in detection of many important analytes including metal ions, bioactive small molecules, drugs, protein, DNA, bacteria, cancer cells and so on with high selectivity and sensitivity [65,66,67,68,69,70,71,72,73,74,75,76,77,78,79,80,81,82,83,84,85,86,87,88,89,90,91,92,93,94,95,96,97,98,99,100,101,102,103,104,105,106,107,108,109,110,111,112,113,114,115,116,117,118,119,120,121,122,123,124,125,126,127,128,129,130,131,132,133,134,135,136,137,138,139,140,141,142,143,144,145,146,147,148,149,150,151,152,153,154,155]. Compared with electrochemical and other approaches, detection of DNA using ECL has the advantages of: (1) extremely low detection limits; (2) no need to use radioactive labels; (3) simple and rapid detection; and (4) the use of relatively more stable ECL labels rather han those employed in chemiluminescence approaches [156,157,158]. Due to the continuous efforts made by researchers all over the world in this field, significant progress has been achieved. Now, ECL measurements are routinely performed in both laboratories and clinics using either homemade or commercialized instruments [157,158]. To date, dozens of excellent reviews on the subject of ECL have been published and readers are directed to these reviews for more comprehensive information [29,80,94,95,157,158,159,160,161,162,163,164,165,166,167,168,169,170,171]. Here, we will mainly discuss the recent progress made in detection of DNA and other important targets with functional nucleic acids after a brief introduction of ECL mechanism. A few early seminal reports will also be included to show the development in a historical view.

### 3.1. Mechanism of ECL

The mechanisms of ECL using Ru(bpy)_3_^2+^ have been extensively studied and well summarized in several reviews [29,157,158]. ECL converts electrochemical energy into light. Specifically speaking, ECL involves a series of reactions taking place upon an electrode, while in the presence of certain analytes, the reactions occurred upon the electrode change, resulting in different light signals.

For ECL using Ru(bpy)_3_^2+^ (or its analogues) as a luminophore, several mechanisms have been proposed, including the “oxidative-reductive” co-reactant pathway, the “reductive-oxidative” co-reactant pathway and the hot electron-induced pathway [29,157,158]. A co-reactant is a species that is oxidized or reduced upon an electrode and thus generating the intermedium which reacts with an ECL luminophore to produce an excited state. Since most analysis follows the “oxidative-reductive” co-reactant pathway, the mechanism is briefly discussed here. For the “oxidative-reductive” co-reactant pathway, the overall general ECL mechanism remains the same for all the co-reactants, though specific reactions may be evolved for each co-reactant. In a typical “oxidative-reductive” co-reactant pathway, Ru(bpy)_3_^2+^ is used as the ECL luminophore and reacts with a co-reactant (in most cases tri-*n*-propylamine, shorten as TPrA) governed by Equations (3)–(6) :

Ru(bpy)_3_^2+^ − e^−^ → Ru(bpy)_3_^3+^ (3)

TPrA − e^−^ →[TPrA·]^+^ → TPrA·+ H^+^ (4)

Ru(bpy)_3_^3+^ + TPrA·→ Ru(bpy)_3_^2+^* (5)

Ru(bpy)_3_^2+^* → Ru(bpy)_3_^2+^ + *hν* (6)

TPrA analogues (such as aliphatic reducing amines), oxalate, and even DNA bases have been used as co-reactants to generate ECL signal. However, for most of the bioanalytical assays discussed in the current review, TPrA was used as the co-reactant unless otherwise specified. As shown in the equations above, Ru(bpy)_3_^2+^ is oxidized to Ru(bpy)_3_^3+^ at the surface of an electrode while TPrA is oxidized to TPrA when a positive potential is applied. Then, Ru(bpy)_3_^3+^ is reduced by TPrA, forming the exited-state Ru(bpy)_3_^2+^*. Ru(bpy)_3_^2+^* is not stable and decays to the ground state, emitting a red light [29,157,158] (note: since most of the bioassays were performed in aqueous solution, water oxidation was involved in the ECL process; though the water oxidation process quenches the ECL, it is negligible when compared with TPrA assisted ECL process (Equations (3)–(6)) [65,156]). With this ECL mechanism in mind, one can easily conclude that the ECL intensity is related to both the concentrations of a luminophore (such as Ru(bpy)_3_^2+^) and a co-reactant (such as TPrA). This also denotes that ECL can be used to detect both Ru(bpy)_3_^2+^ (or other luminophore used) and TPrA (or other co-reactant used). On one hand, when the amount of TPrA is constant, the ECL signal intensity is proportional to the concentration of Ru(bpy)_3_^2+^. Most cases shown below fall into this category where the probes (*i.e.*, DNA) are often conjugated with Ru(bpy)_3_^2+^ (or its analogues). On the other hand, when the concentration of Ru(bpy)_3_^2+^ is constant, the ECL signal intensity is proportional to the concentration of TPrA (or other co-reactants).

### 3.2. ECL Detection of DNA

Due to its high sensitivity and selectivity, detection of DNA using ECL method has drawn much attention from all over the world [72,75,76,100,156,172,173,174,175,176,177,178,179,180]. Table 1 summarizes the performances of selected ECL detection approaches listed in this review.

**Table 1:** DNA detection using ECL method with ruthenium complexes.

ECL Luminophore	LOD (mol/L)	Linear Range (mol/L)	Reference
Ru(bpy)_3_^2+^	1.0 × 10^−13^	2.0 × 10^−13^–2.0 × 10^−9^	[72]
Ru(bpy)_3_^2+^	3.9 × 10^−1^°	3.9 × 10^−9^–1.9 × 10^−7^	[75]
Ru(bpy)_3_^2+^	1.0 × 10^−15^	2.0 × 10^−15^–2.0 × 10^−11^	[100]
Ru(bpy)_3_^2+^	2.0 × 10^−13^	1.0 × 10^−12^–1.0 × 10^−^^6^	[156]
Ru(bpy)_3_-[B(C_6_F_5_)_4_]_2_	—	1.0 × 10^−15^–1.0 × 10^−8^	[175]
Ru(bpy)_3_^2+^	9.0 × 10^−15^	2.4 × 10^−14^–1.7 × 10^−12^	[176]
Ru(bpy)_2_(dcbpy)-NHS	9.0 × 10^−11^	2.7 × 10^−1^^0^–4.0 × 10^−9^	[177]
Ru(bpy)_2_(dcbpy)-NHS	6.7 × 10^−12^	1.7 × 10^−11^–1.7 × 10^−9^	[178]
Ru(bpy)_3_^2+^-NHS	1.2 × 10^−15^	5.0 × 10^−15^–1.0 × 10^−13^	[180]
Ru(bpy)_3_^2+^	1.0 × 10^−15^	1.0 × 10^−14^–1.0 × 10^−11^	[181]
Ru(bpy)_2_(cbpy)-NHS	5.0 × 10^−13^	1.0 × 10^−12^–1.0 × 10^−7^	[182]
Ru(bpy)_3_^2+^	1.0 × 10^−15^	1.0 × 10^−14^–1.0 × 10^−11^	[183]
Ru(bpy)_2_(cbpy)-NHS	1.0 × 10^−13^	1.0 × 10^−13^–1.0 × 10^−9^	[184]
[Ru(bpy)_2_(mcbpy)]^2+^	4.0 × 10^−11^	1.0 × 10^−1^^0^–1.0 × 10^−7^	[185]
Ru(bpy)_3_^2+^	9.1 × 10^−14^	1.0 × 10^−13^–1.0 × 10^−9^	[186]
Ru(phen)_3_^2+^	1.5 × 10^−14^	2.5 × 10^−14^–1.0 × 10^−1^^0^	[187]
[Ru(dmbpy)_2_(PIND)_2_]^2+^	4 × 10^−1^^3^	7 × 10^−1^^3^–4 × 10^−^^10^	[56]

*Notes*: bpy, 2,2'-bipyridine; dcbpy, 4,4'-dicarboxylic acid-2,2'-bipyridine; cbpy, 4-carboxylic acid-2,2'-bipyridine; dmbpy, 4,4'-dimethyl-2,2'-bipyridine; mcbpy, 4-methyl-4'-(carbonylaraino(2-(tert-butoxycarbonylamino)ethyl))-2,2'-bipyridine; phen, 1,10-phenanthroline; B(C_6_F_5_)_4_, tetrakis(pentafluoro-phenyl)borate; PIND, N,N'-bis(3-propyl-imidazole)-1,4,5,8-naphthalene diimide.

#### 3.2.1. DNA as the Co-reactant for Detection

As discussed in the mechanism section, when the concentration of Ru(bpy)_3_^2+^ is constant, the ECL signals can be used to qualify the co-reactant involved. Thorp and others already showed that DNA can be catalytically oxidized by Ru(bpy)_3_^2+^ [44]. Thus when DNA is used as the co-reactant, its amount can be determined by ECL method [75,76,188].

**Figure 6:**
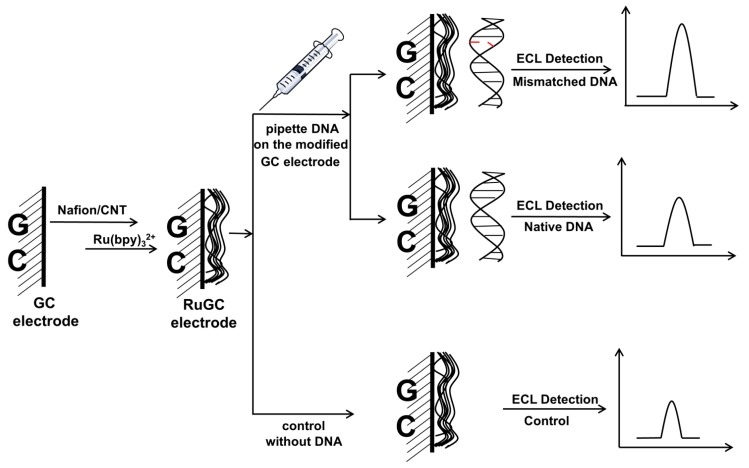
Schematic of ECL DNA detection using a Ru(bpy)_3_^2+^ modified GC electrode. GC, glassy carbon; CNT, carbon nanotubes. Adapted with permission from ref. [75], copyright (2007) Elsevier.

Wei and coworkers described a sensitive label-free ECL DNA detection approach based on catalytic guanine and adenine bases oxidation (guanine bases were mainly involved) with a Ru(bpy)_3_^2+^ modified glassy carbon (GC) electrode (Figure 6 and Equations (7)–(12)) [75]. 

Ru(bpy)_3_^2+^_@CNT/Nafion_ − e^−^ → Ru(bpy)_3_^3+^_@CNT/Nafion_ (7)

Ru(bpy)_3_^3+^_@CNT/Nafion_ + Guanine → Ru(bpy)_3_^2+^_@CNT/Nafion_ + Guanine(-H)^•^ + H^+^ (8)

Ru(bpy)_3_^3+^_@CNT/Nafion_ + Guanine → Ru(bpy)_3_^2+^_@CNT/Nafion_ + Guanine^+•^ (8')

Guanine^+•^ → Guanine(-H)^•^ + H^+^ (8'')

Ru(bpy)_3_^3+^_@CNT/Nafion_ + Guanine(-H)^•^ → Ru(bpy)_3_^2+^*_@CNT/Nafion_ + Guanine_2ox_ (9)

Ru(bpy)_3_^2+^*_@CNT/Nafion_ → Ru(bpy)_3_^2+^_@CNT/Nafion_ + *hν* (10)

Ru(bpy)_3_^2+^_@CNT/Nafion_ + Guanine(-H)^•^ → Ru(bpy)_3_^+^_@CNT/Nafion_ + Guanine_2ox_ (11)

Ru(bpy)_3_^+^_@CNT/Nafion_ + Ru(bpy)_3_^3+^_@CNT/Nafion_ → Ru(bpy)_3_^2+^_@CNT/Nafion_ + Ru(bpy)_3_^2+^*_@CNT/Nafion_ (12)

Ru(bpy)_3_^3+^_@CNT/Nafion_ + Guanine2ox → Ru(bpy)_3_^2+^_@CNT/Nafion_ + Guanine_3ox_ (13)

Ru(bpy)_3_^3+^_@CNT/Nafion_ + Guanine3ox → Ru(bpy)_3_^2+^*_@CNT/Nafion_ + Guanine_4ox_ (14)

Guanine_2ox_ could be 8-oxoguanine or other two-electron oxidized products. Since the two-electron oxidized products are easier to be oxidize by Ru(bpy)_3_^3+^ than guanine itself, the reactions in Equations (13) and (14) could also be involved. However, these species have not been clearly determined and specified in ECL reactions yet. With this method, the authors were able to distinguish dsDNA and their denatured counterparts with a limit of detection (LOD) of 30.4 nM. What’s more, they successfully detected single-base mismatch of p53 gene sequence segment with high sensitivity at a concentration of 0.393 nM. Though the proposed method is highly sensitive and selective, it is a signal-off detection mode. Also, the ECL signals are also closely related with the guanine and adenine bases content in the probe and target DNA. Thus, more widely employed detection approaches are based on Ru(bpy)_3_^2+^-conjugated probes (as discussed below).

#### 3.2.2. Ru(bpy)_3_^2+^-Conjugated Probes for DNA Detection

**Figure 7:**
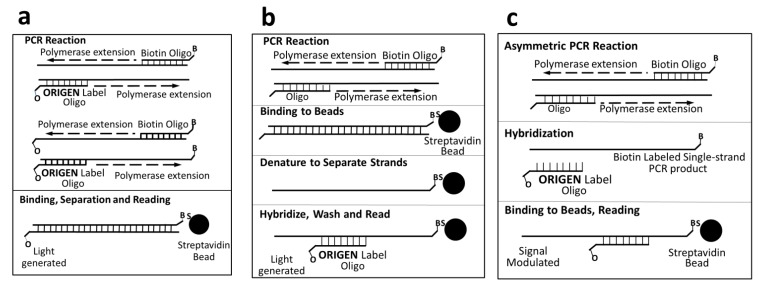
ECL detection of PCR products using Ru(bpy)_3_^2+^ conjugated DNA as probes. (**a**) direct incorporation PCR format with Origen-labeled oligonucleotides and biotin-labeled oligonucleotides as primers; (**b**) usual PCR format but with a biotinylated primer to allow the generation of biotinylated PCR product; (**c**) asymmetric PCR format, generating single-stranded biotinylated DNA for later hybridization to Origen-labeled oligonucleotides. Adapted from ref. [172], copyright (1991) American Association for Clinical Chemistry.

In the presence of a high concentration of co-reactant, such as TPrA, the ECL signal is proportional to the amount of Ru(bpy)_3_^2+^. When Ru(bpy)_3_^2+^ is conjugated with a probe DNA, the conjugates can be used to qualify a target DNA. In 1991, researchers from IGEN, Inc. (Rockville, MD, USA) demonstrated that ECL can be used to qualify polymerase chain reaction (PCR) products of oncogenes, viruses, and cloned genes [156,172]. As shown in Figure 7, for all the three assay formats proposed, Ru(bpy)_3_^2+^ conjugated DNA probes (*i.e.*, Origen Label Oligo) were used to generate ECL signals. Thanks to the PCR amplification and high-sensitivity of ECL, they achieved rapid detection of as low as 10 copies of target. Since these pioneering reports, many formats for ECL DNA detection have been developed [95,189].

**Figure 8:**
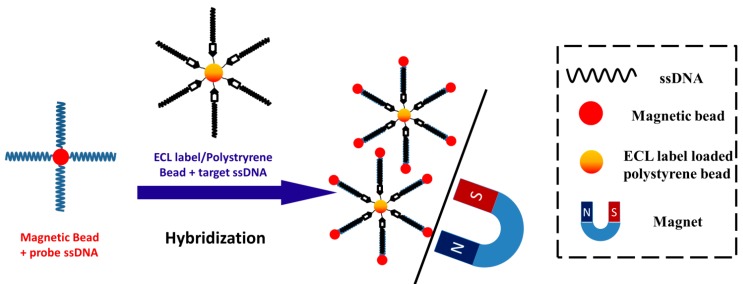
Schematic of two beads mode for ECL DNA detection. Reprinted with permission from ref. [175], copyright (2004) American Chemical Society.

Bard’s group reported the detection of target ssDNA (t-ssDNA) by hybridizing it with probe ssDNA (p-ssDNA), using ECL as the detection signal [68,175]. In the initial study, p-ssDNA was immobilized on an Au electrode and t-ssDNA was conjugated with Ru(bpy)_3_^2+^ (the ratio of t-ssDNA to Ru(bpy)_3_^2+^ was 1:1) [68]. The hybridization of p-ssDNA and t-ssDNA brought Ru(bpy)_3_^2+^ to the electrode surface, thus generating ECL signals. Later, Ru(bpy)_3_-[B(C_6_F_5_)_4_]_2_ loaded polystyrene beads instead of single Ru(bpy)_3_^2+^ were used for DNA labeling, the signal was thus highly amplified due to billions of luminophores were encapsulated in a single bead. As shown in Figure 8, t-ssDNA was conjugated to the surface of polystyrene beads containing the ECL luminophores (*i.e.*, Ru(bpy)_3_-[B(C_6_F_5_)_4_]_2_). Magnetic beads were modified with p-ssDNA to capture and hybridize with t-ssDNA. When the t-ssDNA was complementary with p-ssDNA, they hybridized with each other and brought two kinds of beads together. The hybridized beads can be magnetically separated from reaction solution and reacted with co-reactant TPrA in MeCN, thus the presence of t-ssDNA was reported by the ECL signal. They were able to detect t-ssDNA with a LOD of 1.0 fM and a linear range of 1.0 fM to 10 nM. Besides, this approach was also able to distinguish t-ssDNA from two base pair mismatched ssDNA. However, these approaches may not be very practical since t-ssDNA was directly conjugated to the beads (or Ru(bpy)_3_^2+^). Thus sandwich sensing platforms were later developed for ECL DNA detection.

**Figure 9:**
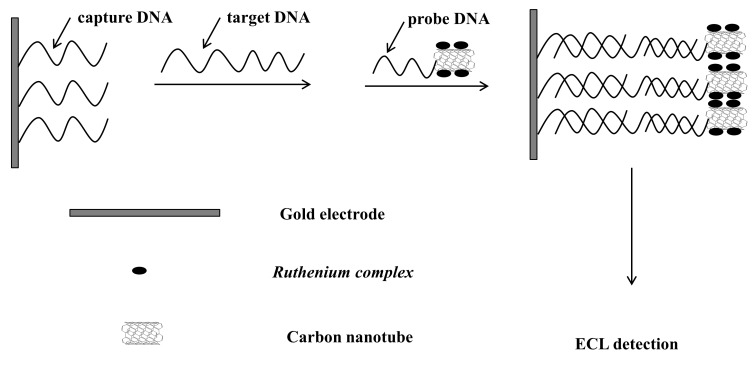
Sandwich sensing platform for ECL DNA detection using probe ssDNA and ruthenium complexes loaded carbon-nanotubes as ECL probes. Reprinted with permission from ref. [176], copyright (2007) Elsevier.

Zhang and coworkers reported an ultrasensitive ECL detection protocol using single-walled carbon-nanotubes (SWNT) loaded with plenty of ruthenium complexes (Figure 9) [176]. A sandwich sensing platform was employed. The p-ssDNA and capture ssDNA (c-ssDNA) were attached to SWNT and Au electrode, respectively. In the presence of t-ssDNA, a sandwich structure was formed and thus a strong ECL response was generated (Figure 9). Again, a large amount of ruthenium complexes were loaded onto SWNT for signal amplification and a detection limit of 9 fM was reported. They also investigated how the modification of the gold electrode affected the sensitivity of ECL detection of DNA [178]. They modified a gold electrode with gold nanoparticles via Au-S bond. Interestingly, they found that after modification the detection limit of t-ssDNA (6.7 pM) was much lower than that on a bare gold electrode (0.12 nM).

**Figure 10:**
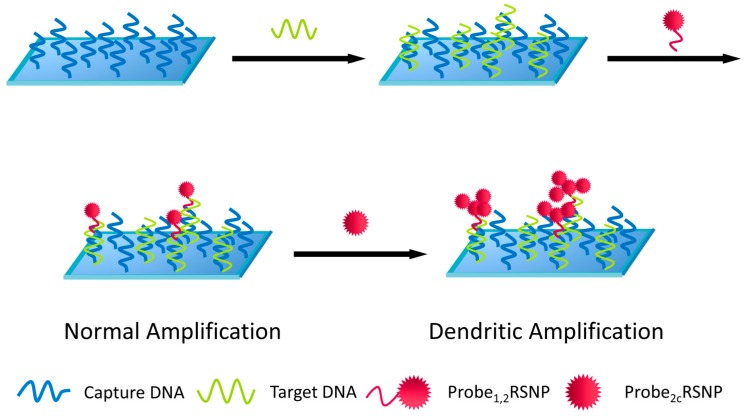
Sandwich DNA detection using dendritic Ru(bpy)_3_^2+^-doped silica nanoparticles for ECL signal amplification. RSNP, Ru(bpy)_3_^2+^-doped silica nanoparticles. Reprinted with permission from ref. [181], copyright (2010) Royal Society of Chemistry.

Besides polystyrene beads and SWNT, other nanomaterials have also been investigated to load (or encapsulate) multiple ECL luminophores for enhanced signaling [181,190]. For example, when Ru(bpy)_3_^2+^ molecules were encapsulated within silica nanoparticles, they can be used for amplified ECL [82,181]. As shown in Figure 10, further amplification can be achieved when dendritic structures of Ru(bpy)_3_^2+^-doped silica nanoparticles were used as ECL labels [181]. As high as 5-fold ECL enhancement was obtained when comparing dendritic structures with single silica nanoparticles, and as low as 1 fM t-ssDNA was detected. 

**Figure 11:**
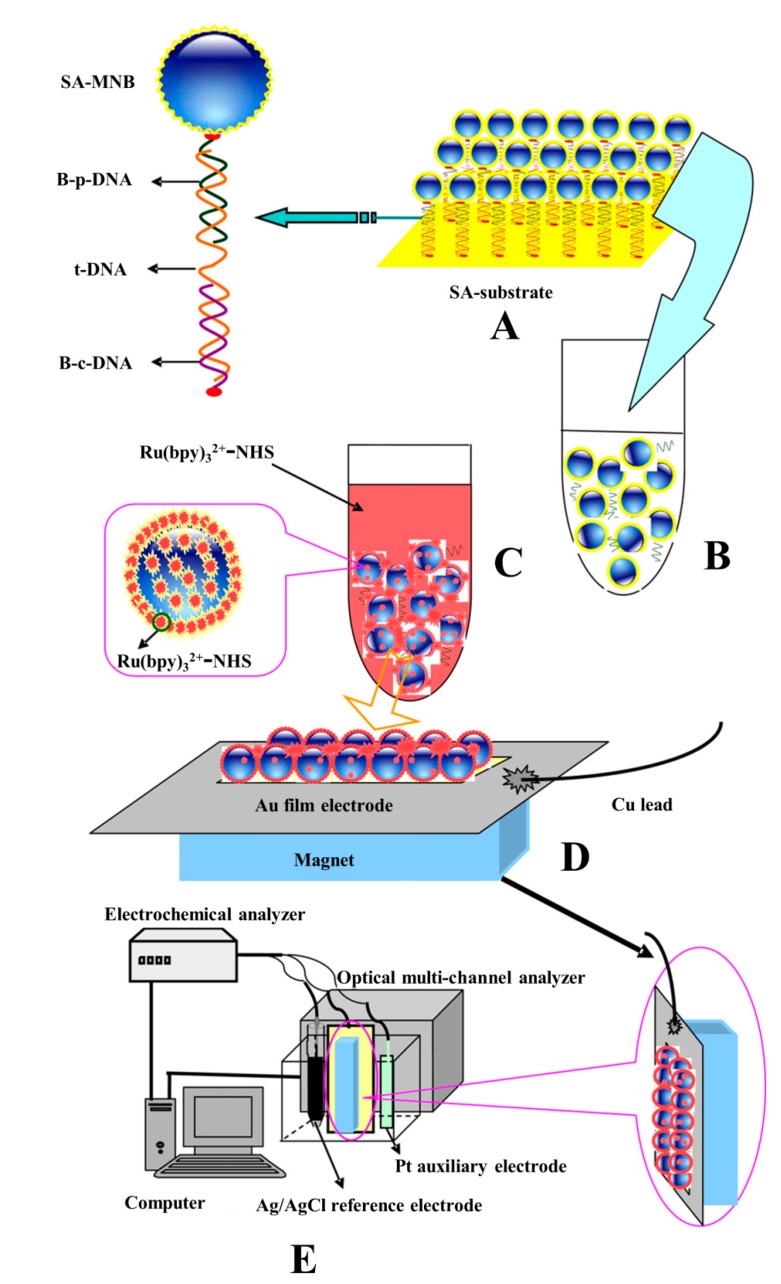
Schematic representation of the process of ECL spectrometry for determination of DNA by a combination of magnetic nanobeads attached with Ru(bpy)_3_^2+^-NHS as labels and overview of the ECL spectrometry detection system. SA-MNB, streptavidin coated magnetic nanobead; B-p-DNA, biotinylated probe DNA; t-DNA, target DNA; B-c-DNA, biotinylated capture DNA; NHS, N-hydroxysuccinimide ester. Reprinted with permission from ref. [180], copyright (2012) Elsevier.

Jin* et al.* reported an ultrasensitive ECL detection approach using streptavidin-coated magnetic nanobeads as the ECL luminophore carrier (Figure 11) [180]. Due to the large specific surface area of the magnetic nanobeads, multiple ECL luminophores conjugated with streptavidin were coated onto the nanobeads and thus enhanced ECL signals were got. Notably, the luminophore Ru(bpy)_3_^2+^-NHS (NHS is N-hydroxysuccinimide ester) was post-conjugated as shown in Figure 11. Their approach was highly sensitive and realized a detection limit as low as 1.2 fM. The β-2-microglobulin gene from human breast cancer cells was also successfully evaluated using the proposed method. 

**Figure 12:**
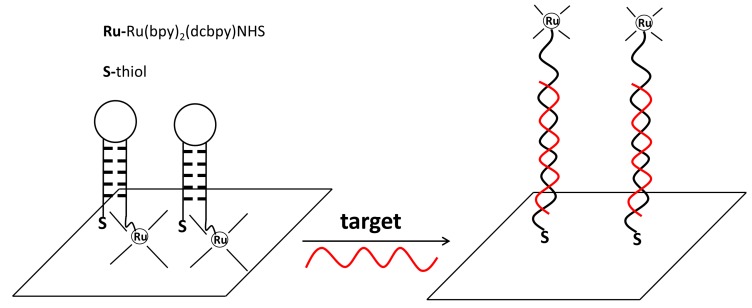
Schematic of a hairpin-DNA probe for DNA detection. Reprinted with permission from ref. [177], copyright (2008) American Chemical Society.

Besides the sandwich assay format, molecular beacon like format was also adopted for ECL DNA detection [177,182]. For instance, Zhang* et al.* designed a highly selective ECL biosensor to detect t-ssDNA using hairpin DNA as the recognition element (Figure 12) [177]. The hairpin-DNA probe was dually labeled at both ends with thiol and Ru(bpy)_3_^2+^-NHS ester, respectively. When the pre-folded hairpin-DNA probe was attached onto an Au electrode via Au-S bond, strong ECL signals were generated due to the proximity of Ru(bpy)_3_^2+^ to the electrode surface. However, when t-ssDNA was added to the system, the stem-loop of the hairpin-DNA probe was converted to a linear double-helix conformation, making Ru(bpy)_3_^2+^ move far from the electrode surface and thus causing the ECL signal decrease. With this platform, they achieved a LOD of 0.09 nM and single-base mismatched t-ssDNA discrimination.

**Figure 13:**
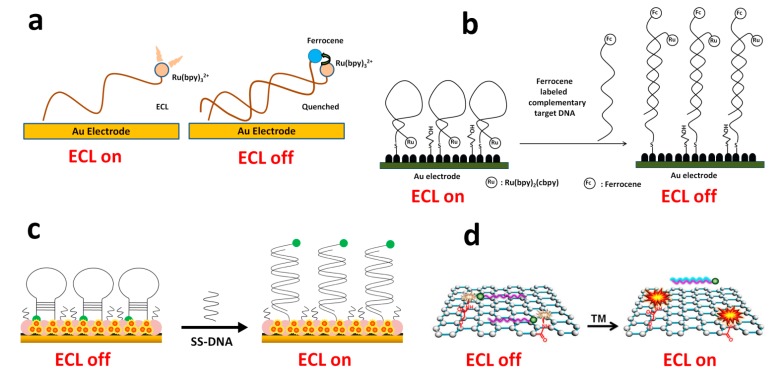
ECL DNA detection based on quenching the ECL of Ru(bpy)_3_^2+^ by ferrocene. (**a**) reprinted with permission from ref. [191], copyright (2006) American Chemical Society; (**b**) reprinted with permission from ref. [184], copyright (2012) Elsevier; (**c**) reprinted with permission from ref. [183], copyright (2010) Elsevier; (**d**) reprinted with permission from ref. [185], copyright (2014) American Chemical Society.

Cao* et al.* found that ferrocene could quench the ECL of Ru(bpy)_3_^2+^ [191]. Based on this interesting phenomenon, they developed an assay for DNA detection (Figure 13a and Equations (15) and (16)). Since then, numerous studies have employed similar strategy for DNA detection [183,184,185,192]. For example, when a molecular beacon like probe DNA conjugated with Ru(bpy)_3_^2+^ was attached onto Au nanoparticles assembled electrode, enhanced ECL signal was obtained compared with bare Au electrode due to larger surface areas of Au nanoparticles (Figure 13b) [184]. In the presence of t-ssDNA conjugated with ferrocene, the hybridization brought Ru(bpy)_3_^2+^ and ferrocene together and thus the ECL of Ru(bpy)_3_^2+^ was quenched. With this design, lower than 0.1 pM t-ssDNA was detected. Instead of tagging Ru(bpy)_3_^2+^ onto a probe DNA, Wang* et al.* immobilized Ru(bpy)_3_^2+^ onto an electrode surface and developed a signal-on detection strategy as shown in Figure 13c [183,192]. Cui and coworkers recently reported an interesting homogeneous signal-on protocol to detect rpoB genes of Mycobacterium tuberculosis (Figure 13d) [185]. The probe DNA labeled with ferrocene quenched the ECL of Ru(bpy)_3_^2+^ pre-immobilized onto graphene. In the presence of t-ssDNA, it formed duplex with ferrocene labeled probe DNA. Due to the weaker interaction between duplex DNA and graphene, the formed duplex was released from graphene, which led to the recovery of ECL signal. The method had high selectivity and could distinguish single-base mismatch from complementary t-DNA. It had a LOD of 40 pM. Other quenchers, such as pristine carbon nanotubes, were also explored as ECL quenchers for DNA detection [186].

ferrocene (Fc) − e^−^ → ferrocenium (Fc^+^) (15)

Ru(bpy)_3_^2+^* + ferrocenium → Ru(bpy)_3_^3+^ + ferrocene (16)

#### 3.2.3. Label-free ECL DNA Detection

**Figure 14:**
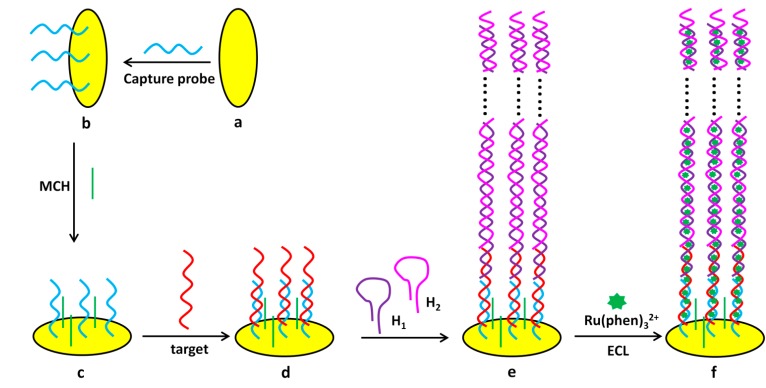
ECL DNA detection using as Ru(phen)_3_^2+^ as ECL luminophore. The signal was amplified through* in situ* hybridization chain reaction. MCH, mercaptohexanol. Reprinted with permission from ref. [187], copyright (2012) American Chemical Society.

Besides the above detection protocols using luminophore conjugated probe DNA, label-free sensing platforms also attracted a lot of attention. Most of the label-free assays make use of the intercalation interactions between DNA and ruthenium complexes [110,173,174,187,193]. Tris(1,10-phenanthroline) ruthenium(II) (Ru(phen)_3_^2+^) is a duplex DNA semi-intercalator with high ECL performance. As shown in Figure 14, amplified with* in situ* hybridization chain reaction, highly sensitive and selective DNA detection was achieved by using ECL signals from Ru(phen)_3_^2+^. Single-base match could be detected with the proposed method. A linear range from 25 fM to 100 pM and a LOD of 15 fM were obtained [187]. 

### 3.3. ECL Detection of Other Targets with Functional Nucleic Acids

Functional nucleic acids, such as aptamers and DNAzymes, are emerging recognition elements for biosensing and bioanalysis [194,195,196,197,198,199,200,201]. As alternatives to antibodies and enzymes, functional nucleic acids have been used to detect wide range of important targets from metal ions, small molecules, protein, to cancer cells [195,196,197,198,199,202,203,204,205,206]. In this section, the use of ECL as detection method for fabricating functional nucleic acids biosensing systems is discussed.

#### 3.3.1. Ru(bpy)_3_^2+^-conjugated Functional Nucleic Acids as Probes for Detection

**Figure 15:**
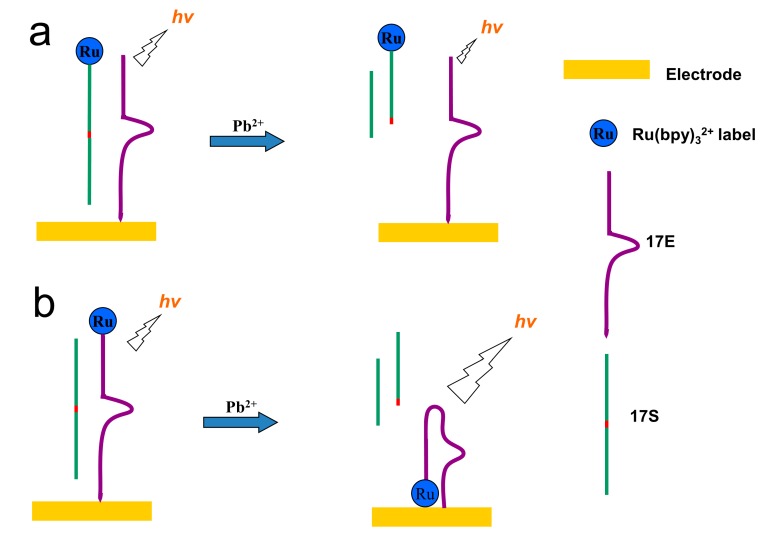
DNAzyme based lead ions detection using as Ru(bpy)_3_^2+^ as ECL luminophore. (**a**) signal-off strategy; (**b**) signal-on strategy. (**a**) adapted with permission from ref. [207], copyright (2009) Royal Society of Chemistry; (**b**) adapted with permission from ref. [208], copyright (2011) Elsevier.

Using lead ions specific DNAzyme as a model system, researchers have developed sensitive and specific ECL assay for lead ions [207,208]. As shown in Figure 15, both signal-off and signal-on detection strategies have been developed. When Ru(bpy)_3_^2+^ was labeled on the substrate DNA strand, the lead-induced cleavage of the substrate DNA gave the decreased ECL signal (Figure 15a). On the other hand, when Ru(bpy)_3_^2+^ was labeled on the enzyme DNA strand, the cleavage increased ECL signal due to the decreased distance between Ru(bpy)_3_^2+^ and the electrode surface. Due to the intrinsic sensitivity of ECL method, LODs of 0.1 nM and 1.4 pM were obtained for signal-off and signal-on methods, respectively, which were better than usually used fluorescent, colorimetric and electrical methods [207,208]. The methods also showed high specificity towards lead ions over other competing metal ions, such as Cu^2+^, Zn^2+^, Mn^2+^, Co^2+^, Ca^2+^, Hg^2+^,* etc.* Based on the very specific thymine-Hg-thymine interaction, an ECL approach for mercury ions detection was also reported [93].

**Figure 16:**
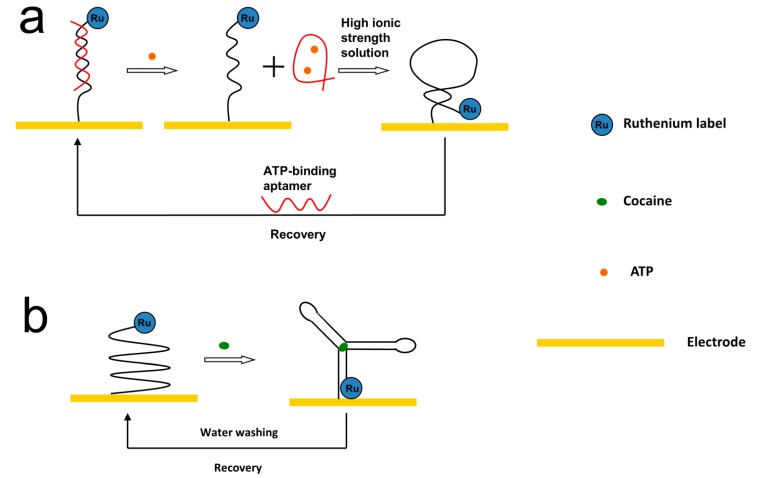
Reusable ECL aptasensor for small molecules detection using Ru(bpy)_3_^2+^ as ECL luminophore. (**a**) adapted with permission from ref. [87], copyright (2009) Elsevier; (**b**) adapted with permission from ref. [209], copyright (2010) American Chemical Society.

Using the structure switch strategy, reusable ECL aptasensors for small molecule detection were developed (Figure 16) [87,209]. In Yao and coworkers’ report, an ATP binding aptamer was immobilized onto an electrode by hybridizing with its partially complementary c-ssDNA, which was labeled with Ru(bpy)_3_^2+^ (Figure 16a) [87]. The ATP presented in the sample bound to the aptamer and released the aptamer from the hybridized duplex. The capture ssDNA then formed a hairpin structure and brought Ru(bpy)_3_^2+^ closer to the electrode, resulting in stronger ECL signals. The aptasensing platform could be easily recovered by re-hybridizing with the aptamer. Miao’s group employed a slight different design for cocaine detection (Figure 16b) [209]. The two ends of the aptamer were labeled with amine (5') and Ru(bpy)_3_^2+^ (3'), respectively. The labeled aptamer was attached on a 4-aminobenzene sulfonic acid pre-treated electrode through cross-linking between 4-aminobenzene sulfonic acid and 5'-amine of the aptamer. Cocaine would bound to the aptamer and induce a conformational change, which brought Ru(bpy)_3_^2+^ in proximity to the electrode, resulting in stronger ECL signals. The fabricated aptasensor could be recovered by simply washing with water. Besides the high reusability, the aptasensor also possessed as long as three weeks storage stability [209].

**Figure 17:**
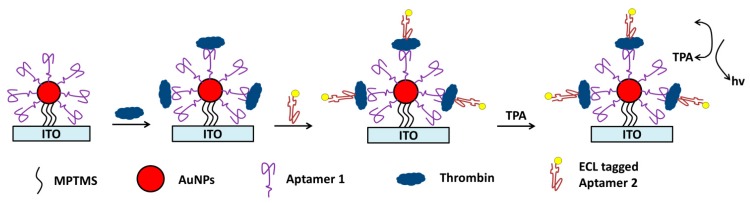
ECL aptasensor for α-thrombin detection using Ru(bpy)_3_^2+^ as ECL luminophore. MPTMS, (3-mercaptopropyl)trimethoxysilane; ITO, indium tin oxide; TPA, tri-*n*-propylamine; AuNPs, gold nanoparticles. Reprinted with permission from ref. [81] copyright (2008) Elsevier.

Fang and coworkers reported an ECL aptasensing platform for α-thrombin detection (Figure 17) [81]. α-thrombin has two aptamers, one is 15-mer in length while the other is 29-mer. In their report, 15-mer aptamer modified Au nanoparticles (immobilized on an electrode) were used as capture probes and Ru(bpy)_3_^2+^ conjugated 29-mer aptamer as signaling probes. The presence of α-thrombin induced the formation of sandwich structures, thus giving ECL signals. The method had a LOD of 10 nM. Because of the high selectivity of the aptamer used, the aptasensor had negligible response towards β-thrombin and γ-thrombin. With the same sandwich strategy, Li* et al.* used Ru(bpy)_3_^2+^ loaded SWNT to conjugate probe aptamer for signal amplification [210]. They achieved a much lower LOD for thrombin (*i.e.*, 3 fM). 

**Figure 18:**
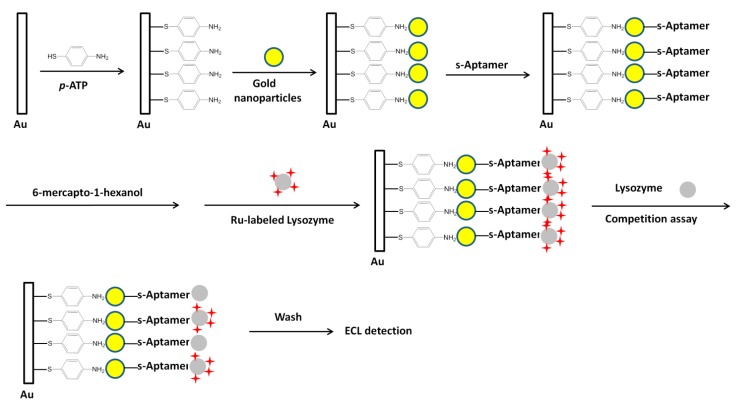
ECL aptasensor for lysozyme detection using Ru(bpy)_3_^2+^ as ECL luminophore. *p*-ATP, *p*-aminothiophenol. Reprinted with permission from ref. [84], copyright (2008) John Wiley and Sons.

As shown in Figure 18, Bai* et al.* developed a competing strategy for lysozyme detection [84]. The Ru(bpy)_3_^2+^ conjugated lysozyme molecules were first bound to the pre-immobilized aptamer on an electrode. This gave high ECL signals. The presence of target lysozyme competed against the Ru(bpy)_3_^2+^ conjugated lysozyme, leading to a signal decrease. Through the signal-off mode, they got a linear range from 0.1 aM to 10 pM and a LOD of 0.1 aM towards lysozyme detection. 

**Figure 19:**
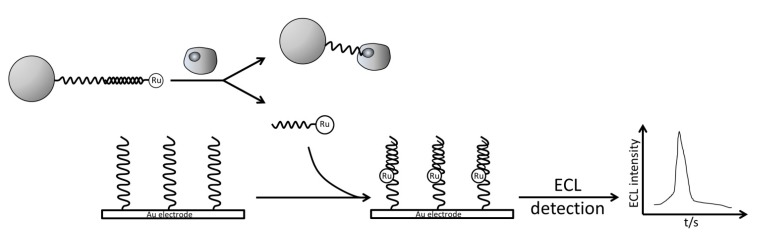
ECL aptasensor for cancer cells detection using Ru(bpy)_3_^2+^ as ECL luminophore. Reprinted with permission from ref. [90], copyright (2010) John Wiley and Sons.

Cancer cells were also successfully detected using their specific aptamers via the ECL method [90,211]. In Ding and coworkers’ report, the Ru(bpy)_3_^2+^ labeled p-ssDNA was initially immobilized onto a magnetic bead by forming duplex with Ramos cancer cell specific aptamer. Ramos cells would compete for binding to the aptamer against the p-ssDNA, leading to the release of the p-ssDNA. Then, the Ru(bpy)_3_^2+^ labeled p-ssDNA was detected in an Au electrode via ECL method (Figure 19). With the established method, Ramos cells were detected with a linear range of 100 to 3,000 cell/mL and a LOD of 89 cells/mL. Yu* et al.* used the same strategy but introduced Au nanoparticles onto both magnetic beads and electrode for amplification [211]. They were able to achieve a lower LOD of 78 cells/mL for Ramos cells.

**Figure 20:**
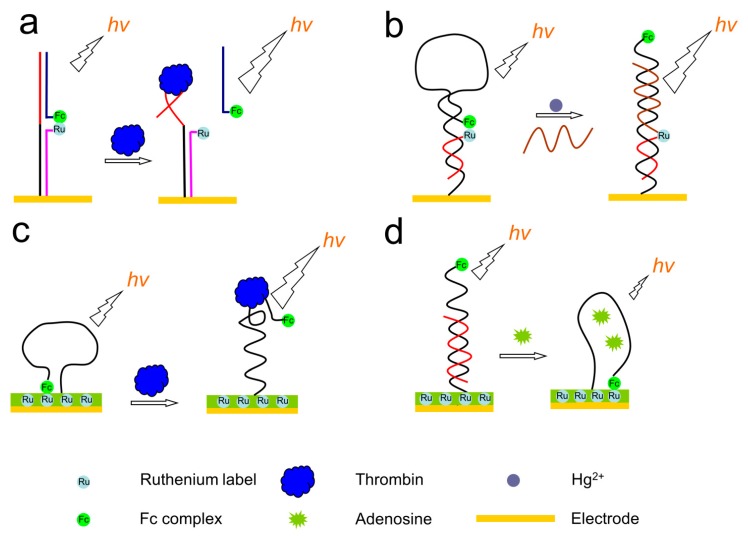
ECL sensing platforms based on the quenching of Ru(bpy)_3_^2+^ ECL by ferrocene. (**a**) adapted with permission from ref. [212], copyright (2008) Elsevier; (**b**) adapted with permission from ref. [213], copyright (2013) Elsevier; (**c**) adapted with permission from ref. [214], copyright (2009) Elsevier; (**d**) adapted with permission from ref. [215], copyright (2010) Elsevier.

The quenching phenomenon of Ru(bpy)_3_^2+^ ECL by ferrocene was also explored to construct functional nucleic acids based sensing systems [191,212,213,214,215,216,217]. As shown in Figure 20, most of these types of sensing platforms adopted a signal-on strategy. Usually, Ru(bpy)_3_^2+^ and ferrocene were brought together on an electrode, leading to the quenching of Ru(bpy)_3_^2+^ ECL. The presence of a target would bound to the aptamer through a structure switch and thus separate ferrocene away from Ru(bpy)_3_^2+^, resulting in the recovery of Ru(bpy)_3_^2+^ ECL. For instance, Zhang’s group reported mercury ions and thrombin sensing platforms based on the designs shown in Figure 18a,b [212,213]. Similar approaches were adopted by Ye* et al.* to detect adenosine [216]. Instead of conjugating Ru(bpy)_3_^2+^ to p-ssDNA, Fang’s group immobilized Ru(bpy)_3_^2+^ onto the electrode surface (Figure 18c,d) [214,215]. The pre-assembled aptamers with hairpin structures on the electrode, which were labeled with ferrocene, quenched the ECL of Ru(bpy)_3_^2+^. The presence of targets such as thrombin and adenosine removed ferrocene from the electrode and recovered the ECL signals. Besides ferrocene, other quenchers, such as SWNT, were also used to construct such type of sensors. For instance, a sensitive ECL aptasensor for adenosine based on SWNT quenching of Ru(bpy)_3_^2+^ ECL was reported by Li* et al.* [218].

#### 3.3.2. Label-Free Method

**Figure 21:**
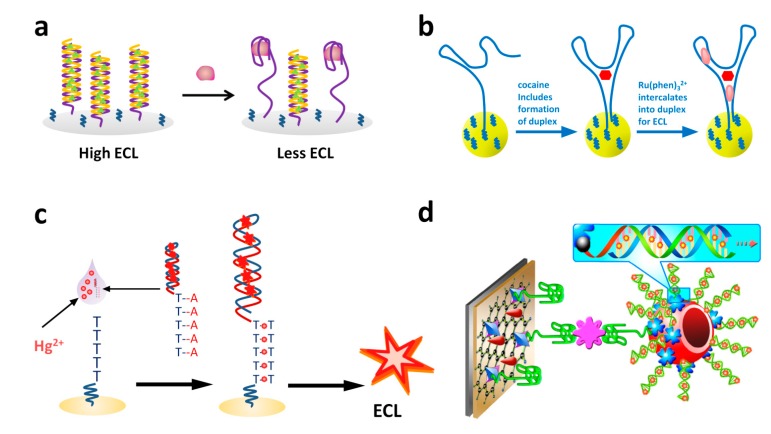
Label-free ECL aptasensing platforms using Ru(phen)_3_^2+^ as ECL luminophore. (**a**) signal-off platform for protein detection; (**b**) signal-on platform for small molecules (such as cocaine) detection; (**c**) signal-on platform for mercury ions detection; (**d**) signal-on platform for thrombin detection. (**a**) reprinted with permission from ref. [219], copyright (2009) American Chemical Society; (**b**) reprinted with permission from ref. [221], copyright (2011) Royal Society of Chemistry; (**c**) reprinted with permission from ref. [220], copyright (2010) Royal Society of Chemistry; (**d**) reprinted with permission from ref. [225], copyright (2013) Elsevier.

As the same case of label-free ECL DNA sensors, label-free ECL aptasensors based on interactions (such as intercalation and electrostatic interactions) between DNA and ruthenium complexes also received much attention recently [88,219,220,221,222,223,224,225]. For most of the studies, Ru(phen)_3_^2+^ was used as a semi-intercalator to react with duplex DNA and thus gave ECL signals [219,220,221,222,224,225]. Adopted the strategy shown in Figure 21a, Yin’s group reported the detection of thrombin and lysozyme [219,222]. The duplex of the aptamer and its partially complementary ssDNA was pre-assembled onto an electrode, which interacted with Ru(phen)_3_^2+^ and gave ECL signals. The presence of target released the complementary ssDNA and thus decreased the amount of Ru(phen)_3_^2+^ interacted, leading to ECL signal decrease. With the proposed method, extremely low detection limit (*i.e.*, 0.2 attomolar in mass, 0.05 pM) towards thrombin was obtained. The LOD of lysozyme was 0.45 pM. The proposed aptasensor also possessed many other merits, such as high selectivity, low-cost, broad-spectrum practicability. They also reported a label-free ECL aptasensor for cocaine detection based on target induced conformational change strategy (Figure 21b) [221]. Cocaine induced the formation of duplex structures, which could interact with Ru(phen)_3_^2+^ and gave ECL signals. Interestingly, Ru(phen)_3_^2+^ and its semi-intercalated duplex DNA as a whole could be used as ECL luminophore for assay (Figure 19c,d) [220,225]. Yin and coworkers reported a mercury ions sensor based on such design, which could detect femtomole level of mercury ions (Figure 21c) [220]. A supersandwich-type aptasensor for thrombin detection was developed by Gui* et al.*, in which Ru(phen)_3_^2+^ interacted duplex DNA structures were assembled onto hollow Au nanoparticles for signal amplification (Figure 21d) [225]. Through the amplification, excellent sensitivity was obtained with a LOD of 1.6 fM.

**Figure 22:**
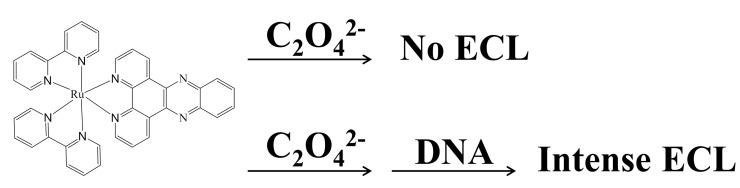
Scheme of ECL switch, based on [Ru(bpy)_2_dppz]^2+^ and DNA. Reprinted with permission from ref. [88], copyright (2009) American Chemical Society.

Xu and coworkers investigated the ECL switch behavior of [Ru(bpy)_2_dppz]^2+^ (dppz is dipyrido[3,2-*a*:2',3'-*c*]phenazine) for the first time [88]. Using oxalate as the co-reactant, they showed that ECL of [Ru(bpy)_2_dppz]^2+^ was negligible, which was attributed to the quenching effect of water. The triplet metal-to-ligand charge transfer excited state could be quenched when water and the phenazine nitrogen formed hydrogen bondings. When [Ru(bpy)_2_dppz]^2+^ intercalated into duplex DNA, the hydrogen bondings were broken due to shielding effect of DNA. The intercalation thus resulted in an ECL enhancement about 1,000 times (Figure 22) [88]. Based on this remarkable enhancement, they developed a signal-off aptasensor for ATP. The aptasensor showed good selectivity against UTP, CTP, and GTP. The LOD was 100 nM and the linear range was from 0 to 1 μM.

**Figure 23:**
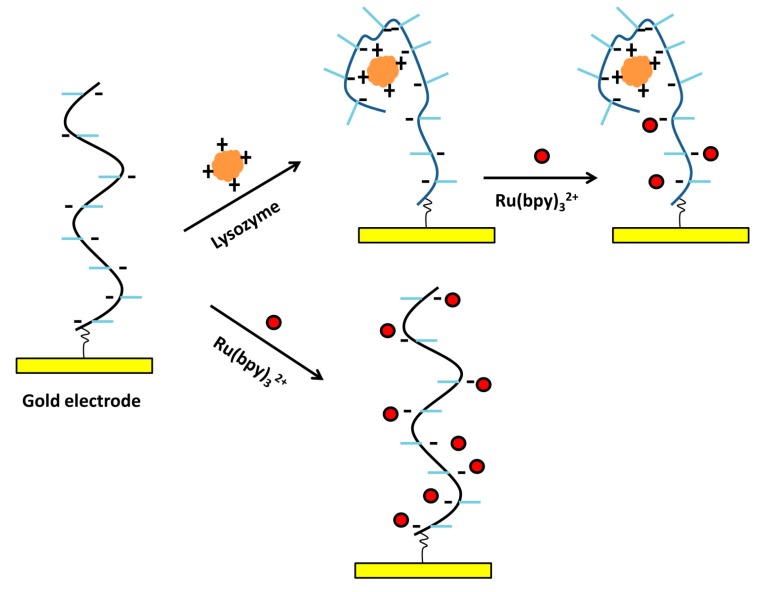
Label-free ECL sensing platforms for lysozyme determination using Ru(bpy)_3_^2+^ as luminophores. Reprinted with permission from ref. [223], copyright (2011) Elsevier.

Besides the intercalation interactions with ruthenium complexes with duplex DNA, the electrostatic interactions between ruthenium complexes and DNA were also used to design aptasensors [223]. For instance, positively-charged Ru(bpy)_3_^2+^ would interact with lysozyme binding aptamer and thus gave strong ECL signals. The presence of positively-charged lysozyme would form complex with its aptamer and partially neutralized the aptamer’s negative charges. This would cause the dissociation of Ru(bpy)_3_^2+^ from the aptamer and the decrease of ECL signals (Figure 23). A 0.12 pM LOD was obtained for the proposed sensing format [223].

## 4. Photoluminescent Methods

**Figure 24:**
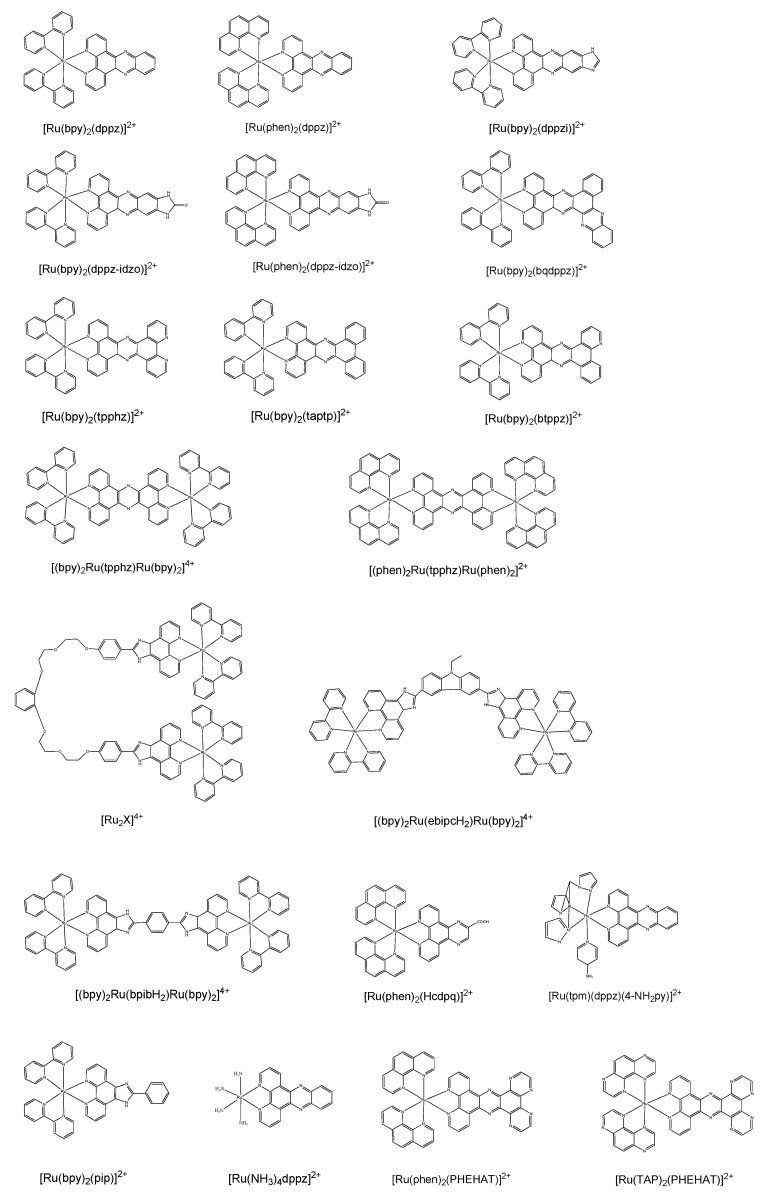
Structures of selected ruthenium complexes as “light switch” probes.

Barton* et al.* found that [Ru(bpy)_2_(dppz)]^2+^ showed almost no photoluminescence in aqueous solution but emitted intense photoluminescence after adding calf thymus DNA. The enhancement factor of luminescence intensity was more than 10,000-fold. Hence, when DNA was added to aqueous solution containing [Ru(bpy)_2_(dppz)]^2+^, the luminescence yield increased dramatically. This phenomenon was denoted as the “light switch” effect [226]. Since Barton’s group reported the application of [Ru(bpy)_2_(dppz)]^2+^ as a “light switch” molecule for dsDNA, great interest has been focused on designing ruthenium complexes as photoluminescent probes (both “light switch” and other types) for bioanalysis including DNA detection [226,227,228,229,230,231,232,233,234,235,236,237,238,239,240,241,242,243,244,245,246,247,248,249,250,251,252,253,254,255,256,257,258,259,260,261,262,263,264]. Till now, researchers have reported many “light switch” molecules like [Ru(phen)_2_(dppz)]^2+^ for DNA analysis (Figure 24) [264,265,266,267,268,269,270,271,272,273,274,275,276,277,278].

### 4.1. Ruthenium Complexes as “Light Switch” Effect Probes

#### 4.1.1. Mechanism of “Light Switch” Effect

Since the report of “light switch” effect by Barton’s group, many efforts have been put into exploring its mechanism [267,268,270]. It is widely believed that the “light switch” effect derives from hydrogen bond formation between ruthenium complexes and the protic solvent (such as the water molecules in aqueous solutions), which leads to the excited-state luminescence quenching. For instance, there are two phenazine nitrogen atoms in the ligand dppz of [Ru(bpy)_2_(dppz)]^2+^, which are apt to interact with water molecules, leading to the emission quenching [226,265,266,279,280,281]. However, in the presence of dsDNA, the dppz ligand of [Ru(bpy)_2_(dppz)]^2+^ is intercalated into DNA duplex and the emission is recovered. This assumption was further confirmed by crystallographic studies, indicating that ruthenium complexes intercalate the double-helix structure of DNA through the direction of DNA minor groove [275,276,282,283]. The remarkable luminescent difference between the quenched and excited states of ruthenium complexes makes them ideal candidates for photoluminescence detection of DNA.

Carrying out experiments on [Ru(phen)_2_(dppz)]^2+^, McGarvey* et al.* found that the “light switch” effect was also observed in the presence of ssDNA when [Ru(phen)_2_(dppz)]^2+^ was either covalently attached onto or simply mixed with ssDNA [245]. They conducted transient resonance Raman and time-resolved steady-state luminescence measurements. Interestingly, they found that luminescence intensity was dependent on the ssDNA length and at least six bases were required for the “light switch” effect. They proposed a slightly different mechanism. It is suggested that as long as the water molecules were excluded from the dppz ligand by forming a protective “cavity” with ssDNA added, the “light switch” effect could be observed. The deep intercalation of the dppz ligand into the DNA bases may not be necessary for the enhanced luminescence. 

#### 4.1.2. Factors Affecting the “Light Switch” of Ruthenium Complexes

Several critical factors affecting the “light switch” efficiency of ruthenium complexes are discussed in this section. Ligands of ruthenium complexes play critical roles in their “light switch” performance. A previous report showed that [Ru(phen)_2_(dppz)]^2+^ exhibited higher “light switch” efficiency than [Ru(bpy)_2_(dppz)]^2+^ [284]. More, [Ru(NH_3_)_4_(dppz)]^2+^ did not show any photoluminescence in the presence of dsDNA [241]. These results, combined with others, suggested that ancillary ligands with larger conjugated planar ring would result in improved “light switch” efficiency (*i.e.*, NH_3_ << bpy < phen) [240,241,251,284]. The comparison study of bpy and its derivatives as ancillary ligands for ruthenium complexes showed that the “light switch” efficiency followed the order of bpy < 4,4'-dimethyl-2,2'-bipyridine (dmb) < 4,4'-di-t-butyl-2,2'-bipyridine (dtbb). This indicated that hydrophobic ancillary ligands may favor “light switch” [255].

Usually, two bidentate ligands are used as ancillary ligands for ruthenium based “light switch” probes. Recently, it showed that a tridentate and a monodentate ligands together can also be used to construct “light switch” ruthenium probes [229,285,286,287]. For example, the tridentate ligand tpy (tpy is tris(pyrazolyl)methane) and a monodentate N-donor ligand were employed to synthesize [Ru(tpy)(L)(dppz)]^2+^ complexes [229]. It found that the complex coordinated with a pyridine as the monodentate ligand exhibited typical “light switch” behavior in the presence of DNA. However, when the pyridine ligand was replaced by its derivative,* i.e.*, 4-aminopyridine, the “light switch” effect was inhibited significantly at room temperature. It was proposed that the presence of amino group in the monodentate ligand had steric hindrance, preventing the intercalation of dppz into DNA [229].

Besides the effect of ancillary ligands, the “light switch” can also be tuned by changing the intercalating ligand [228,240,288]. For the complexes of [Ru(phen)_2_L]^2+^, the effects of different ligands (*i.e.*, dppz, HAT, and PHEHAT) were examined. (Note, HAT is 1,4,5,8,9,12-hexaazatriphenylene; PHEHAT is 1,10-phenanthrolino[5,6-b]1,4,5,8,9,12-hexaazatriphenylene.) The “light switch” efficiencies of the three complexes followed the order of dppz > HAT > PHEHAT [288]. The weakest emission of [Ru(phen)_2_(PHEHAT)]^2+^ in the presence of dsDNA was attributed to the nature of the complexes rather than the binding affinity towards dsDNA. More interestingly, when the ancillary ligand of [Ru(phen)_2_(PHEHAT)]^2+^ was changed to TAP (TAP is 1,4,5,8-tetraazaphenanthrene), the emission of [Ru(TAP)_2_(PHEHAT)]^2+^ is insensitive to water and could emit 635 nm light. The emission is from the Ru(II) → TAP metal-to-ligand charge transfer (MLCT) rather the Ru(II) → PHEHAT MLCT. The above examples demonstrated that both ancillary and intercalating ligands play key roles in the “light switch” effects of ruthenium complexes, which are determined by the nature of the ligands used. 

When the two ruthenium metal centers are connected by a bridging ligand, the formed binuclear ruthenium complexes may interact with dsDNA via threading intercalation [246,289,290]. Compared with ruthenium monomer, such as [Ru(phen)_2_(dppz)]^2+^, the bridged complex [μ-(11,11'-bidppz)(phen)_4_Ru_2_]^4+^ (note, 11,11'-bidppz is 11,11'-bi(dipyrido[3,2-a:2',3'-c]phenazinyl)), showed similar quantum yield (*i.e.*, similar “light switch” capability). However, the bridged complex exhibited slower DNA association and dissociation rates, making them as ideal drug candidates.

As mentioned above, solvent plays critical roles in the “light switch” of ruthenium complexes. The change of a protic solvent to an aprotic solvent can also create strong luminescence, thus achieving the “light switch” [226,291]. It showed that the solvent used may have different solvation capabilities towards the ruthenium complexes, which then can lead to different emission [252]. Turro* et al.* reported the pronounce solvent effect on the emission of [Ru(bpy)_2_(dppp2)]^2+^ (dppp2 is pyrido-[2',3':5,6]pyrazino[2,3-f][1,10]phenanthroline) [292]. Compared with the 653 nm emission in CH_2_Cl_2_, [Ru(bpy)_2_(dppp2)]^2+^ in CH_3_CN exhibited an emission at 752 nm. More, the 653 nm emission was 19 times stronger. The solvent effect was probably due to the different low-lying MLCT excited states [292].

Temperature-dependent studies were carried out to elucidate the effects of temperature on the “light switch” phenomena and the mechanism [229,267,271]. Meyer, Papanikolas, and coworkers investigated the temperature-dependent excited-state lifetime of [Ru(bpy)_2_(dppz)]^2+^, concluding that the “light switch” of [Ru(bpy)_2_(dppz)]^2+^ from the competition between dark state favorite energetic factors and bright state favorite entropic factors [267]. As mentioned above, the “light switch” of [Ru(tpy)(L)(dppz)]^2+^ complexes was affected by the monodentate ligand used [229]. Thomas and coworkers further demonstrated that the “light switch” was also temperature dependent [229]. For [Ru(tpy)(pyridine)(dppz)]^2+^, its binding affinity towards dsDNA was high, and the change of temperature from 10 to 35 °C did not affect the binding significantly. For [Ru(tpy)(4-aminopyridine)(dppz)]^2+^, it is a weaker dsDNA binder compared with [Ru(tpy)(pyridine)(dppz)]^2+^. The isothermal calorimetry, viscosity and luminescence measurements suggested that [Ru(tpy)(4-aminopyridine)(dppz)]^2+^ bound to dsDNA through intercalation at low temperature (*i.e.*, 10 °C), thus resulting in “light switch”. However, at temperatures of 25 °C and above, the enhanced dsDNA deformation and flexibility would favor groove binding rather that intercalation, which made [Ru(tpy)(4-aminopyridine)(dppz)]^2+^ non-emissive even in the presence of dsDNA [229].

**Figure 25:**
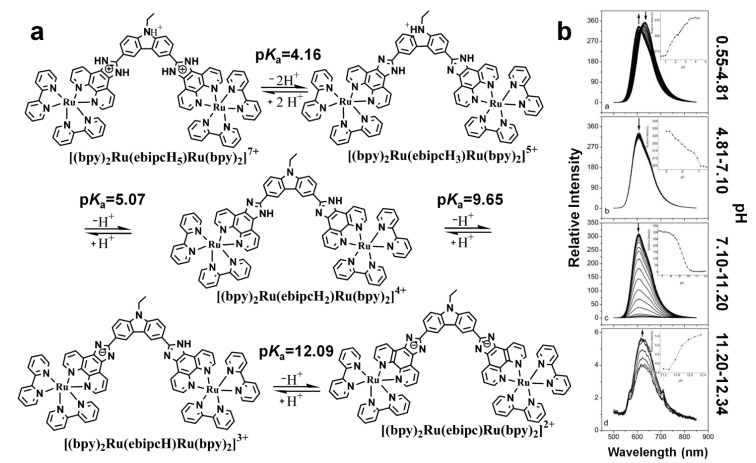
pH effect on “light switch” of [(bpy)_2_Ru(ebipcH_2_)Ru(bpy)_2_]^4+^. (**a**) the acid-base equilibria of [(bpy)_2_Ru(ebipcH_2_)Ru(bpy)_2_]^4+^; (**b**) the changes of emission spectra of [(bpy)_2_Ru(ebipcH_2_)Ru(bpy)_2_]^4+^ upon raising the pH. Reprinted with permission from ref. [238], copyright (2004) American Chemical Society.

Many other factors, such as pH, ionic strength, and quenching species, could also affect the “light switch” of ruthenium complexes [227,235,238,239,256,271]. Since the ligands are critical to the “light switch”, the pH induced ligand structural changes can also affect the “light switch” as expected [227,238,239]. For example, the emission of a dinuclear complex [(bpy)_2_Ru(ebipcH_2_)Ru(bpy)_2_]^4+^ (ebipcH_2_ is N-ethyl-4,7-bis([1,10]-phenanthroline[5,6-f]imidazol-2-yl)carbazole) was sensitive to pH changes (Figure 25) [238]. The bridging ligand ebipcH_2_ has 4 p*K*_a_ values. As the pH increased, the ebipcH_2_ deprotonated sequentially. When the pH changed from 8 to 10, as high as 100 on-off ratio was observed.

#### 4.1.3. Probing DNA Conformational Changes by Light Switchable Ruthenium Complexes

**Figure 26:**
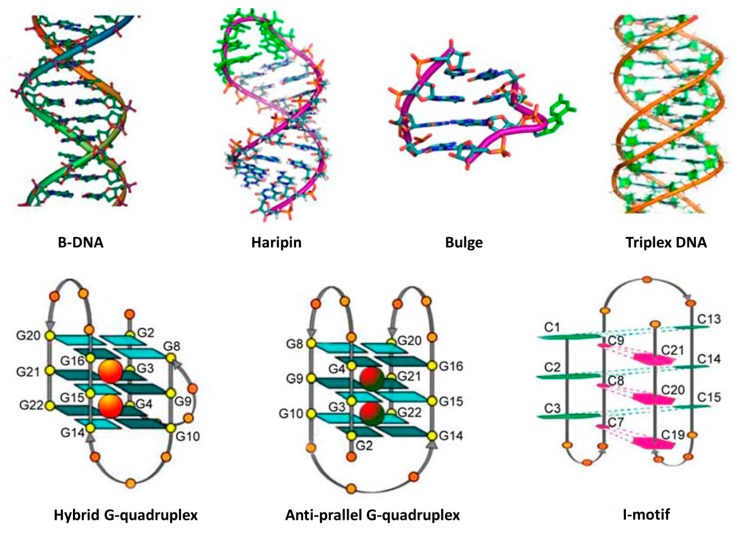
Selected secondary structures of DNA. Reprinted with permission from ref. [263], copyright (2014) Science China Press.

Barton’s group investigated the interactions between dsDNA and two classical complexes,* i.e.*, [Ru(bpy)_2_(dppz)]^2+^ and [Ru(phen)_2_(dppz)]^2+^. They found that the “light switch” effects of these two complexes were strongly dependent on the sequence of DNA [226,265,266,293,294,295]. Further studies indicated that light switchable ruthenium complexes were good luminescent probes to investigate DNA conformational changes (Figure 26) [231,232,233,234,235,236,237,240]. As already discussed, McGarvey and coworkers found that ssDNA (length ≥ 6 bases) could also induce the luminescence of [Ru(phen)_2_(dppz)]^2+^ [245]. They suggested that as long as the intercalating ligand dppz was protected from water molecules, the “light switch” effect could be observed. Choi* et al.* showed that both [Ru(bpy)_2_(dppz)]^2+^ and [Ru(phen)_2_(dppz)]^2+^ could bind to poly(dT*dA-dT) triplex [296]. Compared with dsDNA, the triplex DNA exhibited higher luminescent upon binding to the ruthenium complexes. It suggested that the base triplets in triplex had larger surface area and better protection of the intercalating ligand dppz from water [266].

Besides the ssDNA, dsDNA and triplex DNA discussed above, a lot of efforts have been focused on quadruplex DNAs and their interactions with light switchable ruthenium complexes. Under physiological environments, some G-rich sequences have been proved to form G-quadruplex structures* in vitro* [297,298,299,300,301]. However, the existence of G-quadruplex structure* in vivo* is still under debate [302,303,304,305]. Thus, G-quadruplex detection is of great importance and remains a challenging task. Ji and coworkers explored the “light switch” effects of the two classic complexes,* i.e.*, [Ru(bpy)_2_(dppz)]^2+^ and [Ru(phen)_2_(dppz)]^2+^, with G-quadruplexes and i-motif (their structures are shown in Figure 26) [231,233]. For both [Ru(bpy)_2_(dppz)]^2+^ and [Ru(phen)_2_(dppz)]^2+^, it showed that their binding affinities towards several DNA conformations followed the order of G-quadruplex > dsDNA > i-motif [233]. Thus, the light switching effects followed the same order of G-quadruplex > dsDNA > i-motif. In fact, [Ru(bpy)_2_(dppz)]^2+^ only served as molecular “light switch” in the presence of G-quadruplexes and dsDNA but did not trigger significant “light switch” effect in the presence of i-motif. For the G-quadruplex structures, the mixed hybrid G-quadruplex (in the presence of K^+^) showed better “light switch” performance than the anti-parallel G-quadruplex (in the presence of Na^+^) when using both [Ru(bpy)_2_(dppz)]^2+^ and [Ru(phen)_2_(dppz)]^2+^ as luminescent probes. For the two ruthenium complexes, [Ru(phen)_2_(dppz)]^2+^ showed stronger luminescence than [Ru(bpy)_2_(dppz)]^2+^ in the presence of G-quadruplex [231,233]. This is probably due to the effect of intercalating ligands as discussed above (Section 4.1.2).

Thomas* et al.* explored the binding between dsDNA and dinuclear ruthenium complex [(phen)_2_Ru(tpphz)Ru(phen)_2_]^4+^ (tpphz is tetrapyrido[3,2-*a*:2',3'-*c*:3'',2''-*h*:2''',3'''-*j*]phenazine) (see Figure 24) [237]. Additionally, they found that the complex also had high affinity to G-quadruplex DNA. What’s more, the binding of G-quadruplex DNA to the complex resulted in a distinct blue-shift “light switch” effect.

However, most of ruthenium monomers, such as [Ru(bpy)_2_(dppz)]^2+^ and [Ru(phen)_2_(dppz)]^2+^, showed weak selectivity towards G-quadruplex against dsDNA [231,233,235,237]. Therefore, great efforts have been devoted to develop highly selective “light switch” probes for G-quadruplex by introducing novel intercalating ligands [30,232,234,236,240]. For example, Chao* et al.* synthesized an asymmetric molecular probe,* i.e.*, Ru[(bpy)_2_(bqdppz)]^2+^ (bqdppz is benzo[j] quinoxalino[2,3-h]dipyrido[3,2-*a*:2',3'-*c*]-phenazine) (see Figure 24) [234]. It argued that the design of bqdppz ligand was based on the following concerns: first, high binding affinity of a ruthenium complex towards its target DNA (in this case a G-quadruplex DNA) usually leads to high selectivity; second, the environmental changes after binding (*i.e.*, the ligand protection provided by DNA binding) can affect the “light switch” efficiency. Taking Ru[(bpy)_2_(dppz)]^2+^, a good DNA binder, as the parent probe, the dppz ligand was replaced by bqdppz to prepare a highly selective probe towards G-quadruplexes [234]. The bqdppz could be regarded as an extended dppz by replacing one benzene ring of dppz with a phenazine ring and adding a benzene ring on the other side of the added phenazine ring (see Figure 24). Due to steric hindrance from the asymmetric shape and extended surface area of bqdppz, the as-prepared Ru[(bpy)_2_(bqdppz)]^2+^ could not intercalate into dsDNA effectively, thus exhibiting excellent selectivity towards various G-quadruplexes against dsDNA. It also suggested that the complex should stack on the ends of G-quadruplexes. Surprisingly, the “light switch” responses of Ru[(bpy)_2_(bqdppz)]^2+^ were also dependent on the structures of G-quadruplexes investigated. Compared with other G-quadruplexes, the ruthenium complex had the highest luminescent selectivity toward a human telomeric sequence AG_3_(T_2_AG_3_)_3_ in the presence of potassium ions. AG_3_(T_2_AG_3_)_3_ adopted the (3+1) hybrid G-quadruplex motif. A dramatic enhancement of luminescence was observed when the (3+1) hybrid G-quadruplex was added, which was 45 times brighter than adding of dsDNA in luminescence intensity.

By replacing the dppz ligand with dppz-idzo (dppz-idzo is dppz-imidazolone), Yao and coworkers demonstrated that Ru[(bpy)_2_(dppz-idzo)]^2+^ was an outstanding probe for selective detecting G-quadruplex DNA [236,240]. It showed about 300-fold enhancement in the presence of G-quadruplex DNA in K^+^ solution. The studies indicated that Ru[(bpy)_2_(dppz-idzo)]^2+^ could not only stabilize formed G-quadruplex but also induce the formation of an anti-parallel G-quadruplex. Zhou* et al.* developed a dinuclear ruthenium complex (*i.e.*, [Ru_2_X]^4+^ in Figure 24), which was highly selective for detecting G-quadruplex DNA [232]. They chose high ionic strength systems to imitate the physiological environment and different G-quadruplex sequences to form different structures in the presence of K^+^. They found that the luminescence intensity weakly increased when adding dsDNA and even decreased in the presence of ssDNA. However, the ruthenium complex exhibited significant luminescence enhancement in the presence of G-quadruplex DNA. As pointed out, the self-luminescence of the ruthenium complex was easily detectable. To lower background signal, they chose iodide ions, a luminescence quenching agent that can quench the luminescence of dissociative molecules due to its heavy atom effect, to eliminate self-luminescence of [Ru_2_X]^4+^. With this strategy, duplex DNA and quadruplex DNA were easily distinguished with naked eyes under UV light irradiation.

**Figure 27:**
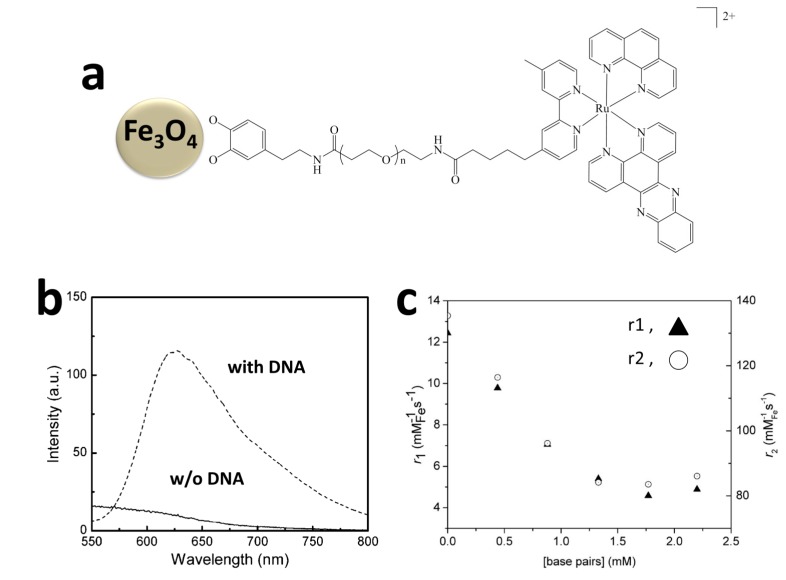
Magnetoluminescent light switch probe for dual mode DNA detection. Reprinted with permission from ref. [306], copyright (2013) American Chemical Society.

Combining the “light switch” effect of [Ru(bpy')(phen)(dppz)]^2+^ and the magnetic relaxivity of iron oxide nanoparticle, Smolensky* et al.* prepared a magnetoluminescent light switch probe for dual mode DNA detection (Figure 27) [306]. Around 20-fold luminescence enhancement was observed for the probe in the presence of DNA. Additionally, both longitudinal and transverse relaxivities of the probe decreased in the presence of DNA. Such dual-functionality probes may find other applications such as catch-and-release purification of DNA besides DNA detection in the future [306].

#### 4.1.4. Bioanalysis Based on the “Light Switch” Effect

**Figure 28:**
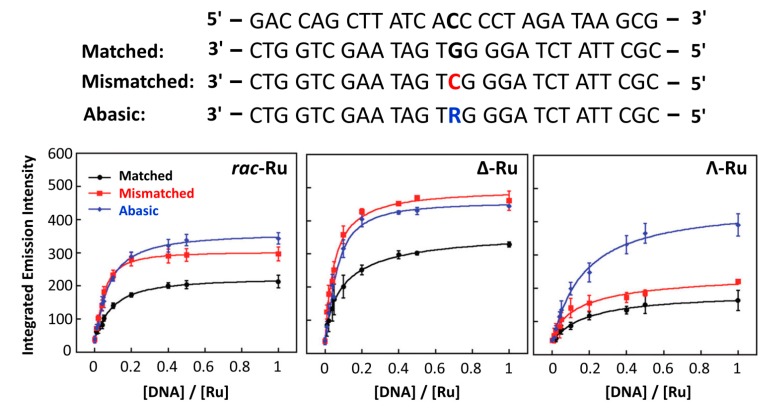
DNA detection with Ru[(bpy)_2_(dppz)]^2+^. Reprinted with permission from ref. [294], copyright (2009) American Chemical Society.

Since most of the light switchable ruthenium complexes can distinguish dsDNA from ssDNA, they are excellent probes for DNA detection. Barton* et al.* reported the detection of DNA using Ru[(bpy)_2_(dppz)]^2+^ as the “light switch” probe [294]. It showed that the conformation of Ru[(bpy)_2_(dppz)]^2+^ itself played a key role in the selectivity. As shown in Figure 28, Λ-Ru[(bpy)_2_(dppz)]^2+^ exhibited much better bioanalytical performance than Δ-Ru[(bpy)_2_(dppz)]^2+^ and *ras*-Ru[(bpy)_2_(dppz)]^2+^. More, Λ-Ru[(bpy)_2_(dppz)]^2+^ also showed better single-base mismatch distinguishing capability than ethidium bromide (EB) and TO-PRO-3. Λ-Ru[(bpy)_2_(dppz)]^2+^ was also used to successfully detect different mismatches in hairpin DNA. Choi* et al.* later reported a homogeneous assay for DNA detection with Ru[(bpy)_2_(dppz)]^2+^ [247]. Again, single-base mismatches were detected using the established method.

Using functional nucleic acids as biorecognition elements, several analytical platforms have been developed [247,248,249,253]. Based on the highly specific thymine-Hg-thymine interaction, a “light switch” protocol for mercury ions detection was proposed [248]. The method had a dynamic range of 1.0 × 10^−9^–1.5 × 10^−7^ M and a LOD of 3.5 × 10^−10^ M towards Hg^2+^. It also had excellent selectivity against other metal ions tested. Then method was then successfully used to monitor Hg^2+^ in drinking water within 30 min. Based on the similar design, a method for Ag^+^ detection was reported using the specific cytosine-Ag-cytosine interaction [253].

Tysoe* et al.* prepared Ru[(bpy)_2_(thppz)]^2+^ and used it as probes for sensing Cu^2+^ (Figure 24) [242]. Due to “light switch” effect, Ru[(bpy)_2_(thppz)]^2+^ emitted strong luminescence in the presence of dsDNA. However, the addition of Cu^2+^ could form coordination complex between Cu^2+^ and the intercalating ligand thppz, thus quenching the luminescence. Based on this interesting phenomenon, a Cu^2+^ sensor could be developed. Using the same complex Ru[(bpy)_2_(thppz)]^2+^ and design, Liu* et al.* showed that Co^2+^ could also quench the luminescence [243]. They further demonstrated the addition of EDTA could recover the luminescence, thus realizing the chemical control of DNA “light switch”.

### 4.2. Ruthenium Complexes Beyond “Light Switch” Probes

Besides being used as light switchable probes, ruthenium complexes have also been employed as other luminophores and even quenchers for bioanalysis [257,258,259,260,261].

#### 4.2.1. Ruthenium Complexes as Luminophores

**Figure 29:**
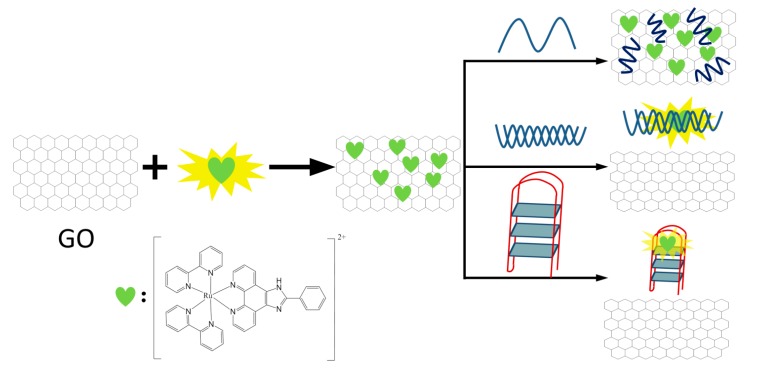
Bioanalysis using Ru[(bpy)_2_(pip)]^2+^ and graphene oxide. Reprinted with permission from ref. [261], copyright (2013) Elsevier.

As shown in Figure 29, Sun* et al.* used graphene oxide and Ru[(bpy)_2_(pip)]^2+^ (pip is 2-phenylimidazo[4,5-f][1,10] phenanthroline) to construct biosensing platforms for DNA and other targets [257]. The luminescence of Ru[(bpy)_2_(pip)]^2+^ was quenched by graphene oxide. Since ssDNA could also adsorb onto graphene oxide, the emission was still inhibited in the presence of ssDNA. However, the presence of target ssDNA would form dsDNA with its complementary ssDNA, thus binding to Ru[(bpy)_2_(pip)]^2+^ and releasing Ru[(bpy)_2_(pip)]^2+^ from the graphene oxide. The luminescence of Ru[(bpy)_2_(pip)]^2+^ was then recovered. Based on the similar strategy, using aptamer as the biorecognition element, K^+^ detection was performed. Later, they showed Hg^2+^ detection could be done making use of the highly specific thymine-Hg-thymine interaction [260]. Since thiols could bind to Hg^2+^ and compete the thymine-Hg-thymine interaction, the sensing platform was also used to evaluate the concentration of biothiols. In principle, any nanomaterials that can quench ruthenium complexes’ luminescence could be used to construct such sensing platform. Metal nanoparticles, carbon nanotubes, graphene, and other 2D nanomaterials should be promising alternatives to graphene oxide. Besides the DNA strand for thymine-Hg-thymine, other functional nucleic acids could also be used for such sensors [257,260,261].

#### 4.2.2. Ruthenium Complexes as Quenchers

**Figure 30:**
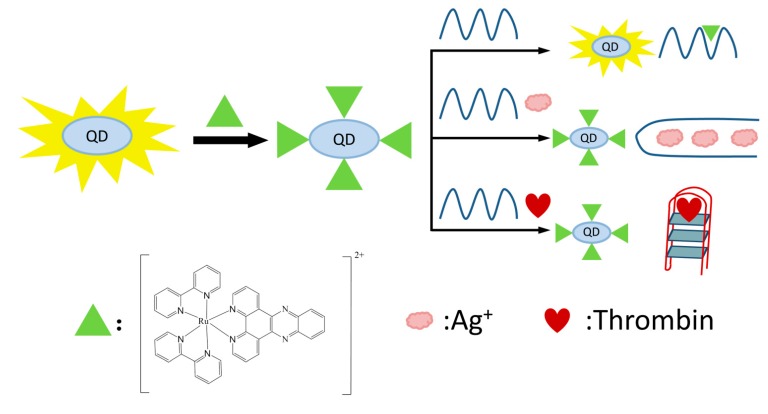
Bioanalysis using Ru[(bpy)_2_(dppz)]^2+^ and quantum dot (QD). Reprinted with permission from ref. [261], copyright (2013) Elsevier.

Researchers found that Ru[(bpy)_2_(dppz)]^2+^ was a good quencher for quantum dot (QD) [258,259,261]. As shown in Figure 30, by exploring this phenomenon, the sensing systems towards metal ions and proteins were developed. For example, the presence of Ag^+^ binding ssDNA recovered the fluorescence of CdTe QD by forming the ssDNA-Ru[(bpy)_2_(dppz)]^2+^ complex [259]. When Ag^+ ^was introduced, it would compete against Ru[(bpy)_2_(dppz)]^2+^ and form complex with the sensing ssDNA, and thus led to CdTe QD's fluorescence quenching. Based on the same design, thrombin detection was achieved by using the corresponding aptamer [258]. As discussed above, other fluorescent nanomaterials and biorecognition elements could be used to fabricate such bioanalytical platforms in future.

## 5. Conclusions and Perspectives

Sensitive and selective biosensing approaches for DNA and other important targets using ruthenium polypyridine complexes as probes have been highlighted in this review. Three widely used detection methods,* i.e.*, electrochemical, ECL and luminescent methods, were discussed with selected examples. They provide us with simple, rapid, selective and sensitive platforms for bioanalysis. In the near future, more efforts could be put into detecting another important nucleic acid, namely, RNA [230]. More, potential applications in cellular sensing and even* in vivo* imaging should be explored since some ruthenium complexes showed interesting two-photon fluorescent properties [272,273,274]. Besides, though commercial instruments based on ECL already have been routinely used in hospitals and laboratories, it could be of great interests if researchers and engineers could develop cheaper, smaller and easier-to-operate commercial devices in the near future [113].
